# Analysis of host microRNA function uncovers a role for miR-29b-2-5p in *Shigella* capture by filopodia

**DOI:** 10.1371/journal.ppat.1006327

**Published:** 2017-04-10

**Authors:** Ushasree Sunkavalli, Carmen Aguilar, Ricardo Jorge Silva, Malvika Sharan, Ana Rita Cruz, Caroline Tawk, Claire Maudet, Miguel Mano, Ana Eulalio

**Affiliations:** 1 Institute for Molecular Infection Biology, University of Würzburg, Würzburg, Germany; 2 UC-BIOTECH, Center for Neuroscience and Cell Biology, University of Coimbra, Coimbra, Portugal; 3 International Centre for Genetic Engineering and Biotechnology, Trieste, Italy; University of Toronto, CANADA

## Abstract

MicroRNAs play an important role in the interplay between bacterial pathogens and host cells, participating as host defense mechanisms, as well as exploited by bacteria to subvert host cellular functions. Here, we show that microRNAs modulate infection by *Shigella flexneri*, a major causative agent of bacillary dysentery in humans. Specifically, we characterize the dual regulatory role of miR-29b-2-5p during infection, showing that this microRNA strongly favors *Shigella* infection by promoting both bacterial binding to host cells and intracellular replication. Using a combination of transcriptome analysis and targeted high-content RNAi screening, we identify UNC5C as a direct target of miR-29b-2-5p and show its pivotal role in the modulation of *Shigella* binding to host cells. MiR-29b-2-5p, through repression of UNC5C, strongly enhances filopodia formation thus increasing *Shigella* capture and promoting bacterial invasion. The increase of filopodia formation mediated by miR-29b-2-5p is dependent on RhoF and Cdc42 Rho-GTPases. Interestingly, the levels of miR-29b-2-5p, but not of other mature microRNAs from the same precursor, are decreased upon *Shigella* replication at late times post-infection, through degradation of the mature microRNA by the exonuclease PNPT1. While the relatively high basal levels of miR-29b-2-5p at the start of infection ensure efficient *Shigella* capture by host cell filopodia, dampening of miR-29b-2-5p levels later during infection may constitute a bacterial strategy to favor a balanced intracellular replication to avoid premature cell death and favor dissemination to neighboring cells, or alternatively, part of the host response to counteract *Shigella* infection. Overall, these findings reveal a previously unappreciated role of microRNAs, and in particular miR-29b-2-5p, in the interaction of *Shigella* with host cells.

## Author summary

MicroRNAs are genome-encoded small non-coding RNAs that play a pivotal role in post-transcriptional regulation of gene expression. In addition to other biological functions, microRNAs are increasingly recognized as important players in the complex interaction between host and bacterial pathogens. In this work we show that microRNAs modulate infection by *Shigella flexneri*, a major causative agent of bacillary dysentery in humans. Specifically, we characterize the dual regulatory role of miR-29b-2-5p during infection, showing that this microRNA strongly favors *Shigella* infection by promoting both bacterial binding to host cells and intracellular bacterial replication. Moreover, we show that miR-29b-2-5p, through the repression of its direct target UNC5C, induces a dramatic increase of host cell filopodia, which are essential to promote bacterial capture and internalization. Importantly, the expression of miR-29b-2-5p is decreased during *Shigella* replication, as a strategy to promote balanced bacterial replication or as part of the host response to counteract infection. This study constitutes the first demonstration that host microRNAs play an instrumental role in the regulation of infection by *Shigella*.

## Introduction

*Shigella flexneri* (*Shigella*), a facultative Gram-negative bacterium, is a major causative agent of bacillary dysentery in humans [1, 2]. A key feature in the pathogenesis of *Shigella* concerns its capability to efficiently colonize the colonic mucosa, in particular the epithelial cells therein, which constitute the primary targets of infection. During the early steps of epithelial cell infection by *Shigella*, bacteria are captured by pre-existing filopodia, which subsequently retract to bring the bacteria in close proximity to the cell body [3]. Although *Shigella* does not express classical adhesins, it was recently shown that the surface exposed autotransporter protein IcsA (also referred to as VirG) exhibits inducible adhesin-like properties upon exposure to bile salts [4]. To enter cells, *Shigella* uses a trigger mechanism, characterized by the formation of actin-rich membrane ruffles that mediate bacteria internalization [5]. Following invasion, *Shigella* escapes from the vacuole and replicates within the host cell cytosol; replication is accompanied by the spreading of *Shigella* to neighboring cells, using actin-based motility [6, 7]. Bacterial effector proteins, injected into the host cytosol through a virulence plasmid encoded type-3 secretion system (T3SS), are essential for all the steps of the interaction of *Shigella* with the host [8–10].

Notwithstanding the ability of *Shigella*, and other bacterial pathogens, to manipulate a vast range of host cellular functions and the multiple mechanisms used by host cells to counteract infection, a still unexplored question regards the interplay between *Shigella* and host cell microRNAs. MicroRNAs (miRNAs) are small RNA molecules, of ca. 22 nt. in length, that play crucial roles in post-transcriptional regulation of gene expression [11]. In mammals, miRNAs act mainly by binding to partially complementary sequences in the 3’ untranslated regions (3’UTRs) or coding sequences of target mRNAs, leading to translation inhibition and/or degradation of target transcripts [12]. The observations that each miRNA targets multiple transcripts, that multiple miRNAs can simultaneously regulate individual transcripts, and the incomplete knowledge of the principles ruling miRNA-target interaction, render the identification of miRNA targets and the assignment of specific phenotypes a complex process [13, 14].

Considering the pervasive role of miRNAs in the control of gene expression [15] and their involvement in numerous pathophysiological processes [11, 16], the premise that miRNAs are relevant players in the interaction between bacterial pathogens and host cells is of particular interest. Indeed, miRNAs have been extensively described as part of the immune response to various bacterial pathogens, from plants to vertebrates, and are increasingly recognized as a novel molecular strategy exploited by bacteria to subvert host cell pathways and thus promote infection [17–19]. Along this line, we have recently shown, through a systematic genome-wide screening approach, that host miRNAs strongly determine the outcome of infection by *Salmonella* Typhimurium [20]. Importantly, functional characterization of selected miRNAs identified through this approach, and of their targets, revealed novel pathways and molecular players crucial to *Salmonella* infection.

Here, we demonstrate that miRNAs modulate infection by *Shigella*, and characterize the dual regulatory role of miR-29b-2-5p during infection, which results from the concomitant regulation of bacterial capture by host cells and of intracellular bacterial replication. Moreover, we show that miR-29b-2-5p through the repression of its direct target UNC5C, a member of the UNC5 netrin-1 receptor family, induces a dramatic increase in the formation of host cell filopodia that are essential for increased bacterial capture and internalization.

## Results

### MiR-29b-2-5p positively regulates *Shigella* infection, by concomitantly increasing binding to host cells and intracellular replication

**Fig 1:**
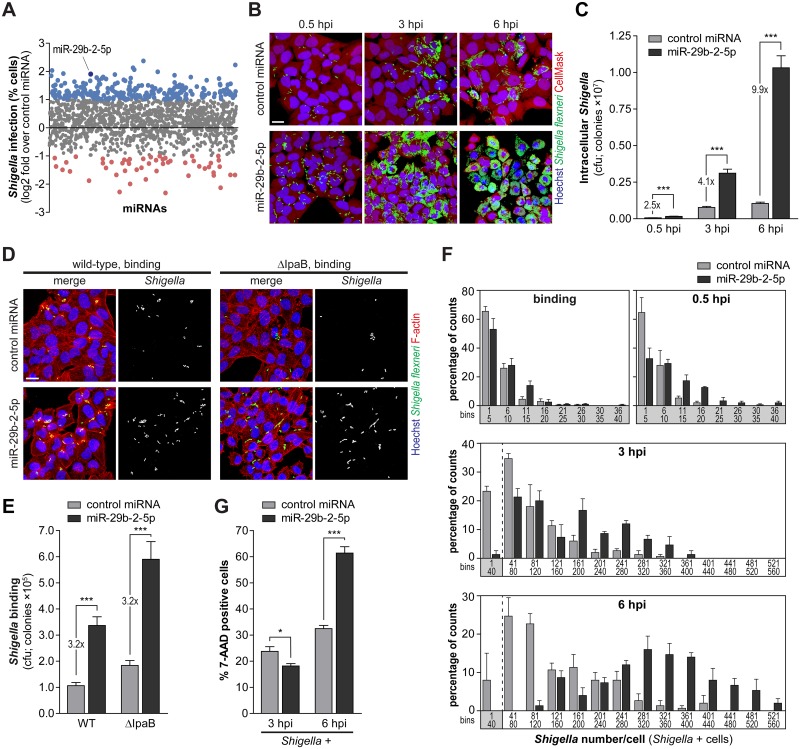
MiR-29b-2-5p favors *Shigella* binding and intracellular replication. A. Effect of human miRNA mimics (genome-wide library of miRNA mimics corresponding to miRBase 19) on the percentage of *Shigella* infected cells (expressed as log2 fold change compared to control miRNA). MiRNAs highlighted in blue and red significantly increase or decrease infection by at least 2-fold, respectively (P<0.05). B and C. Representative images (B) and cfu quantification (C) of intracellular bacteria in HeLa cells infected with *Shigella* WT, upon treatment with miR-29b-2-5p or control miRNA mimics, and analyzed at three times post-infection (0.5, 3 and 6 hpi). Scale bar, 20 μm. D and E. Representative images (D) and cfu quantification (E) of bacteria bound to HeLa cells transfected with miR-29b-2-5p or control miRNA mimics and incubated with *Shigella* WT or ΔIpaB mutant strain for 10 min. Scale bar, 20 μm. F. Distribution of the number of *Shigella* per infected cell at different times post-infection (binding, 0.5, 3 and 6 hpi), in HeLa cells transfected with miR-29b-2-5p or control miRNA mimics. Results are shown for at least 50 infected cells per condition and independent experiment. Values in the X-axis correspond to the extremities of the defined bins. G. Percentage of 7-AAD positive cells following treatment with miR-29b-2-5p or control miRNA mimics for the cell population with internalized *Shigella* (*Shigella* +), analyzed at 3 and 6 hpi. *Shigella* infection was performed at MOI 10, except for the binding experiments (panels D and E) in which MOI 50 was used. Results are shown as mean ± s.e.m. from 4 (panel F), 5 (panels C and E) or 15 (panel G) independent experiments; *P<0.05, ***P<0.001.

To systematically identify miRNAs that modulate infection by *Shigella*, we performed a microscopy-based high-throughput screening of a genome-wide library of miRNA mimics (Fig 1A). Representative images of the 10 highest ranking candidates increasing *Shigella* infection, as well as the percentages of *Shigella* infected cells upon treatment with these 10 miRNAs, are shown in S1A and S1B Fig and S1 Table. Among the miRNAs favoring *Shigella* infection more efficiently, we focused on miR-29b-2-5p, which strikingly increased both the percentage of infected cells and the number of intracellular bacteria per cell (Figs 1A, S1A and S1B).

To validate and further characterize the effect of miR-29b-2-5p on *Shigella*-host interaction, infection was assessed at early, intermediate and late times post-infection (0.5, 3 and 6 hpi, respectively) using three complementary approaches: i) fluorescence microscopy analysis, ii) colony-forming unit (cfu) assays, and iii) qRT-PCR. The results obtained consistently revealed an increase of *Shigella* infection elicited by transfection of miR-29b-2-5p mimic, at the three time points tested (Figs 1B, 1C and S1C). In all experiments, a *C*. *elegans* miRNA (cel-miR-231) was used as negative control; this miRNA has no sequence homology to any known human miRNA. Time-lapse microscopy documenting the dramatic increase of *Shigella* infection by miR-29b-2-5p is included as supplementary material (S1 Video); images extracted at different time points are shown in S2A Fig. It should be noted that for all the experiments (except bacterial binding to host cells), cells were treated with the antibiotic gentamicin to kill the non-internalized bacteria. To confirm the efficacy of the gentamicin treatment, we performed immunofluorescence labelling of *Shigella* before and after a permeabilization step. In contrast to what was observed in permeabilized cells, no labelling with the *Shigella* antibody was detected in non-permeabilized cells, either at early (0.5 hpi) or late (6 hpi) time-points of *Shigella* infection (S1D Fig), confirming that the gentamicin treatment was effective in killing extracellular bacteria.

A significant increase of the fraction of cells with bound bacteria was observed for cells transfected with miR-29b-2-5p mimic (wild-type, WT; Figs 1D, 1E and S2B). This was frequently accompanied by an increase in the formation of actin rich membrane ruffles (Fig 1D), clearly indicating that miR-29b-2-5p promotes a productive interaction of *Shigella* with host cells. To uncouple *Shigella* binding to host cells from bacterial invasion, we performed parallel experiments with the *Shigella* ΔIpaB mutant strain, which binds efficiently to host cells but is impaired in the subsequent steps of invasion [21]. Binding of *Shigella* ΔIpaB to host cells was also increased by miR-29b-2-5p (ΔIpaB; Figs 1D, 1E and S2B), to a similar extent as the wild-type strain, demonstrating that miR-29b-2-5p promotes *Shigella* binding with host cells, prior to the invasion step.

To determine if the increase in *Shigella* infection upon miR-29b-2-5p treatment observed at early times post-infection (2.5-fold at 0.5 hpi) could explain the strong increase observed at later times post-infection (4.1- and 9.9-fold at 3 and 6 hpi, respectively) we performed a time-course experiment using different *Shigella* MOIs (S2C Fig). We observed that infection with MOI 50 results in a 2.6-fold increase of bacterial invasion compared to MOI 10 (0.5 hpi). This difference is comparable to the increase in invasion observed in cells transfected with miR-29b-2-5p mimic in relation to control miRNA (compare Figs 1C and S2C). Interestingly, this difference was maintained at later times of infection (2.3-fold at 3 hpi and 2-fold at 6 hpi, S2C Fig). These results demonstrate that an increase in invasion per se cannot explain the striking increase in bacterial load observed in cells treated with miR-29b-2-5p at later time points, which should thus be ascribed to a cumulative effect of the miRNA on bacterial invasion and intracellular replication. Accordingly, normalization of the results of the cfu experiments at 3 and 6 hpi to the amount of bacteria internalized at the early time point (0.5 hpi) revealed a further increase of bacterial infection at later time points, in cells treated with miR-29b-2-5p (S2D Fig). Moreover, a detailed single-cell analysis of *Shigella* infected cells performed at different time points showed that the number of bacteria bound per infected cell was similar between cells transfected with miR-29b-2-5p and control miRNA (Fig 1F; binding), while at intermediate and late times post-infection the number of bacteria per cell was significantly increased in cells transfected with miR-29b-2-5p (Fig 1F; 3 and 6 hpi). Overall, these results show that miR-29b-2-5p favors two independent steps of *Shigella* interaction with host cells: it promotes binding of bacteria to host cells, and increases *Shigella* intracellular replication.

We observed that miR-29b-2-5p increases infection of the *Shigella* ΔIcsA mutant strain, which is unable to spread to adjacent cells [22, 23], similarly to the wild-type *Shigella* (S3A–S3C Fig), suggesting that the effect of miR-29b-2-5p is not related to spreading. Quantification of the area of *Shigella* infection foci, using automated image analysis, revealed indeed that the average foci size was comparable in miR-29b-2-5p treated cells and control cells (S3D and S3E Fig). *Shigella* ΔIcsA mutant strain was used as control. Of note, infection of cells transfected with miR-29b-2-5p mimic was performed with a lower MOI than that used for control cells, to achieve a comparable level of bacterial invasion. Together, these results exclude the involvement of miR-29b-2-5p in the actin based spreading of *Shigella* to neighboring cells.

When analyzing the fraction of cells containing internalized bacteria (*Shigella* +), we observed that, at late times post-infection, cells transfected with miR-29b-2-5p exhibited significantly higher cell death than cells transfected with a control miRNA (6 hpi; Figs 1G and S2E). These results indicate that the increase of intracellular replication prompted by miR-29b-2-5p decreases viability of the infected cells.

Interestingly, miR-29b-2-5p had no effect on the early or late stages of infection by *Salmonella* Typhimurium, a closely related bacterial pathogen (4 and 20 hpi; S3F–S3H Fig). Binding experiments revealed a mild but significant increase of *Salmonella* binding to host cells treated with miR-29b-2-5p mimic compared to control miRNA (S3I–S3K Fig). Importantly, this effect was clearly less pronounced than that observed in *Shigella* binding experiments (1.3-fold for *Salmonella*, S3I–S3K Fig vs. 3.2-fold for *Shigella*, Figs 1D, 1E and S2B). The binding experiments were performed with *Salmonella* WT and a mutant strain (*Salmonella* Δ4), which binds efficiently to host cells, but it is incapable of triggering membrane ruffling and invasion due to the lack of four key effector proteins (SipA, SopE, SopE2 and SopB) [24–26]. These findings are in full agreement with the results from a systematic analysis of miRNAs modulating *Salmonella* infection that we have performed recently [20]. It should be noted that from the 10 miRNAs that increased *Shigella* infection more efficiently in the screening, only 6 were present in the study by Maudet et al. [20], which was performed with an earlier version of the library of miRNA mimics (988 mimics, miRBase Release 13.0, 2009). Importantly, only one of these miRNAs slightly increased *Salmonella* infection (miR-365, 1.8-fold increase; S1 Table).

### MiR-29b-2-5p levels are decreased upon *Shigella* infection

**Fig 2:**
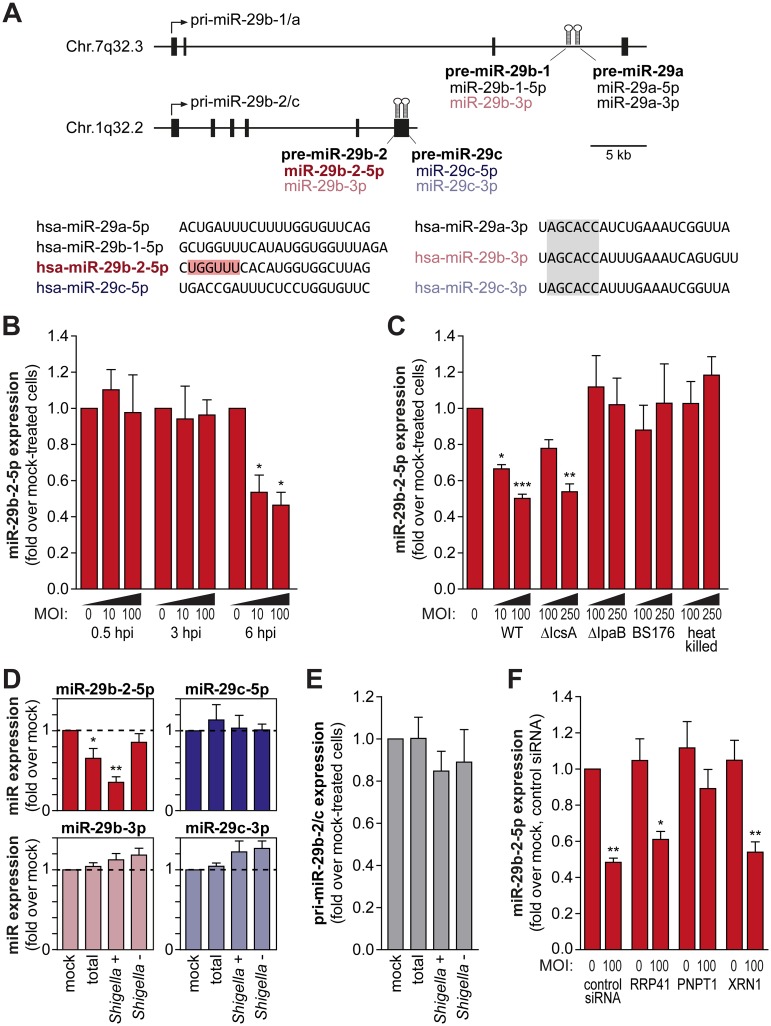
MiR-29b-2-5p is decreased upon *Shigella* infection. A. Schematic representation of the pri-miR-29b-1/a and pri-miR-29b-2/c genomic loci, located respectively on Chr.7q32.3 and Chr.1q32.2. Precursor miRNAs are represented as hairpins in the diagram and exons as black boxes. The sequences of the mature miRNAs derived from the pri-miR-29b-1/a and pri-miR-29b-2/c precursors are shown below the diagram. The unique seed sequence of hsa-miR-29b-2-5p is highlighted in red and the seed sequence common to hsa-miR-29a/b/c-3p is highlighted in gray. B. Levels of mature miR-29b-2-5p in HeLa cells infected with *Shigella* WT (MOI 10 and 100), determined at 0.5, 3 and 6 hpi. C. Mature miR-29b-2-5p levels in HeLa cells infected with *Shigella* WT, ΔIcsA (defective in intercellular spreading), ΔIpaB (able to bind, but invasion deficient) and BS176 (unable to bind and invade) strains, or incubated with heat-killed *Shigella*. Infection was performed at MOI 10 and 100 for *Shigella* WT and MOI 100 and 250 for all other strains, and analyzed at 6 hpi. D. Levels of mature miR-29b-2-5p, miR-29b-3p, miR-29c-5p and miR-29c-3p in the total cell population, *Shigella* + and *Shigella* - fractions, at 6 hpi. HeLa cells were infected with *Shigella* WT expressing GFP at MOI 10 and subjected to cell sorting to separate the population of cells with internalized bacteria (*Shigella* +) and bystander cells (*Shigella* -). E. Expression levels of pri-miR-29b-2/c in the total cell population, *Shigella* + and *Shigella* - fractions, at 6 hpi. F. Levels of miR-29b-2-5p in HeLa cells infected with *Shigella* WT (MOI 100), determined at 6 hpi, in cells transfected with control siRNA or with siRNAs targeting RRP41, PNPT1 or XRN1, which have been involved in mature miRNA degradation. Expression levels of pri-miR-29b-2/c and mature miRNAs was determined by qRT-PCR. Results are normalized to mock-treated cells and shown as mean ± s.e.m. from 3 (panels B, D and E) or 5 (panel C and F) independent experiments; *P<0.05, **P<0.01, ***P<0.001.

Taking into account the strong dependence of *Shigella* infection on the levels of host miR-29b-2-5p, we hypothesized that the levels of this miRNA could be altered during infection. MiR-29b-2-5p originates from the processing of pri-miR-29b-2/c [27], which also results in the production of three other mature miRNAs, specifically miR-29b-3p, miR-29c-5p and miR-29c-3p (Fig 2A). Importantly, miR-29b-2-5p has a unique seed sequence (highlighted in red in Fig 2A), not shared by any of the ca. 2,500 miRNAs currently annotated in the human genome; miR-29b-3p, which has a seed sequence identical to miR-29a-3p and miR-29c-3p, can also originate from a different pri-miRNA (pri-miR-29b-1/a [27]).

qRT-PCR specific for the mature miR-29b-2-5p revealed a decrease in the levels of this miRNA during the course of infection (Fig 2B). The levels of miR-29b-2-5p were significantly decreased at late times post-infection (6 hpi), but remained unchanged at 0.5 and 3 hpi, even at high multiplicity of infection (MOI 100; Fig 2B). Infection with the *Shigella* ΔIcsA mutant strain, invasive and replication competent albeit defective in intercellular spreading [22, 23], reduced miR-29b-2-5p levels similarly to wild-type *Shigella* (Fig 2C), whereas infection with invasion-deficient strains (ΔIpaB and BS176) and incubation with heat-killed *Shigella* did not change miR-29b-2-5p levels (Fig 2C). Moreover, analysis of miR-29b-2-5p levels in sorted populations of cells with internalized *Shigella* (*Shigella* +) and bystander cells (*Shigella* -) at 6 hpi showed that the decrease in miR-29b-2-5p occurs exclusively in the *Shigella* + cell population (Fig 2D). Collectively, these data demonstrate that *Shigella* infection decreases miR-29b-2-5p and that this effect is dependent on bacterial replication and is restricted to cells with internalized bacteria.

To investigate whether *Shigella*-induced regulation of miR-29b-2-5p occurs at the level of miRNA biogenesis or stability, we quantified the levels of the corresponding primary miRNA transcript (pri-miR-29b-2/c; Fig 2A), which remained unchanged upon *Shigella* infection (Fig 2E). Apart from miR-29b-2-5p, the levels of the other mature miRNAs resulting from the processing of pri-miR-29b-2/c remained unchanged (Fig 2D). These results indicate a selective regulation of miR-29b-2-5p at the post-transcriptional level, most likely through modulation of mature miRNA stability.

Although various regulatory mechanisms have been shown to affect miRNA biogenesis and activity, less is known about the regulation of mature miRNA levels through degradation. To evaluate if known miRNA-degrading enzymes are involved in the turnover of miR-29b-2-5p during *Shigella* infection, we tested whether the knockdown of RRP41 (ribosomal RNA-processing protein 41; core component of the exosome complex), PNPT1 (polyribonucleotide nucleotidyltransferase 1, aka PNPase^old-35^) and XRN1 [28], could affect the regulation of miR-29b-2-5p in the context of infection. Interestingly, knockdown of PNPT1 significantly impaired the decrease of miR-29b-2-5p levels in infected cells (Fig 2F), indicating that PNPT1 is involved in the degradation of miR-29b-2-5p during *Shigella* infection. Time course experiments of *Shigella* infection revealed that both bacterial binding and intracellular replication are increased upon PNPT1 knockdown compared to cells treated with a control siRNA (S4A–S4F and S4J Fig). The increase at late times post-infection (6 hpi) can, at least in part, result from a decreased degradation of miR-29b-2-5p in infected cells. However, the increase of infection observed in PNPT1 knockdown cells at the early times post-infection (binding and 0.5 hpi) cannot be explained by changes in the miR-29b-2-5p expression. Moreover, we have analyzed the expression of PNPT1 during the course of infection (0.5, 3 and 6 hpi) and in sorted populations of cells with internalized *Shigella* (*Shigella* +) and bystander cells (*Shigella* -). Neither the PNPT1 mRNA (S4G Fig) nor protein levels (S4H and S4I Fig) were noticeably changed upon *Shigella* infection, indicating that *Shigella* infection is not affecting the expression of PNPT1, but presumably enhancing its exonuclease activity. Overall, these results show that PNPT1 plays an important role in *Shigella* infection, in part through the regulation of miR-29b-2-5p levels.

To determine if decreasing miR-29b-2-5p expression levels affects *Shigella* interactions with host cells, we have applied two experimental approaches: i) transfect cells with a miR-29b-2-5p inhibitor (LNA-based antisense oligonucleotide against mature miR-29b-2-5p); and ii) generate miR-29b-2 knockdown cell lines using CRISPR/Cas9 mediated genome editing [29]. Using these two strategies, we achieved a decrease in miR-29b-2-5p expression of more than two-fold, compared to the control (S5A Fig), which correspond to the levels observed in *Shigella* infected cells at late times of infection (6 hpi; Fig 2B). It should be noted that in the case of miR-29b-2 knockdown cell lines, the expression of the whole precursor miRNA is affected and therefore in addition to miR-29b-2-5p, miR-29b-3p levels were also reduced. The results of both approaches revealed that the interaction of *Shigella* with host cells was impaired upon miR-29b-2-5p knockdown. Specifically, we observed a decrease in binding (S5B–S5D Fig), as well as of intracellular bacteria at 0.5 and 6 hpi (S5E–S5G Fig). The decrease in *Shigella* infection was similar at the binding, invasion (0.5 hpi) and intracellular replication (6 hpi) steps. The lack of a more pronounced effect at 6 hpi (compared to binding or 0.5 hpi) can most likely explained by *Shigella* infection inhibiting miR-29b-2-5p expression (6 hpi, Fig 2B), resulting in a comparable inhibition of replication in control and miR-29b-2-5p knockdown cells at this time point. Together with the data obtained with the miR-29b-2-5p mimic, these results corroborate the concept that miR-29b-2-5p levels are a strong determinant of the extent of *Shigella* infection.

### Multiple miR-29b-2-5p targets are relevant to *Shigella* infection

**Fig 3:**
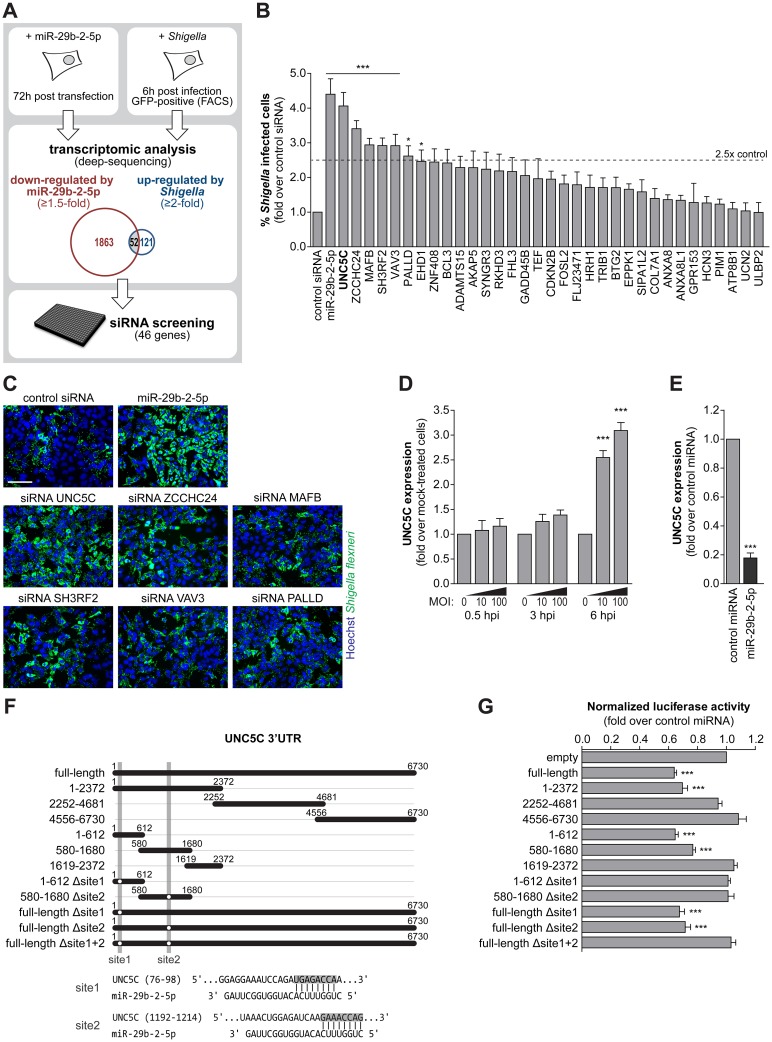
Increase of *Shigella* infection induced by miR-29b-2-5p is dependent on multiple targets. A. Schematic of the workflow for identification of miR-29b-2-5p targets relevant during *Shigella* infection. Transcriptomic analysis revealed fifty-two genes down-regulated by miR-29b-2-5p overexpression (≥1.5-fold) and up-regulated upon *Shigella* infection (≥2-fold, *Shigella* + cell population; 6 hpi). A RNAi screening targeting 46 of these genes was performed to identify siRNAs able to recapitulate the phenotype of miR-29b-2-5p. B. Percentage of HeLa cells infected with *Shigella* WT upon treatment with control siRNA or siRNAs targeting 33 genes, selected as described in Fig 3A. SiRNAs that decreased cell viability to less than 65% of control were excluded. MiR-29b-2-5p is shown for comparison. Results are shown normalized to control siRNA. C. Representative images of HeLa cells infected with *Shigella* WT, upon treatment with the 6 siRNAs that increase percentage of infected cells by at least 2.5-fold, compared with control siRNA. Cells treated with control siRNA and miR-29b-2-5p mimic are shown for comparison. Scale bar, 100 μm. D. UNC5C expression in HeLa cells infected with *Shigella* WT (MOI 10 and 100), at 0.5, 3 and 6 hpi. Results are shown normalized to mock-treated cells. E. UNC5C expression in HeLa cells treated with miR-29b-2-5p mimic. Results are shown normalized to cells transfected with control miRNA mimic. F. Schematic representation of the UNC5C 3'UTR constructs used for the miRNA binding site reporter assays and of the two identified regions of miR-29b-2-5p complementarity to the UNC5C 3’UTR. Shaded regions denote the UNC5C 3’UTR regions deleted in the reporter constructs. G. Results of the luciferase reporter assays. Luciferase activity in cells treated with miR-29b-2-5 is shown compared to control miRNA mimic. For panels B and C, *Shigella* infection was performed at MOI 10 and analyzed at 6 hpi. Results are shown as mean ± s.e.m. from 3 (panel B and E), 4 (panel D), >5 (panel G) independent experiments; *P<0.05, ***P<0.001.

Considering that *Shigella* infection leads to a decrease in miR-29b-2-5p levels, we reasoned that a subset of the genes up-regulated upon infection correspond to derepressed miR-29b-2-5p targets. Therefore, to identify potential direct or indirect miR-29b-2-5p targets that are relevant in the context of *Shigella* infection we compared the genes down-regulated upon miR-29b-2-5p overexpression with those up-regulated at late times of *Shigella* infection (6 hpi; Fig 3A). Transcriptome analysis of HeLa cells transfected with miR-29b-2-5p and control miRNA, performed by deep-sequencing, identified 1,915 putative targets of miR-29b-2-5p (≥1.5 down-regulation; reads ≥25 for cells transfected with control miRNA; Fig 3A). Analysis of *Shigella* infected cells revealed 173 up-regulated genes, compared to mock treated cells (6 hpi; ≥2-fold up-regulation *Shigella* + population relative to mock; ≤ 2-fold *change Shigella* - population relative to mock; reads ≥25 for mock; Fig 3A). Overall, we identified 52 genes that are both repressed by miR-29b-2-5p and have increased expression in *Shigella* infected cells (S2 Table).

To pinpoint, amongst these genes, those relevant to *Shigella* infection, we performed a targeted RNAi screening by individually knocking down 46 genes, for which siRNAs were available (Fig 3A, S3 Table). Knockdown of 6 genes (UNC5C, ZCCHC24, MAFB, SH3RF2, VAV3 and PALLD) increased the percentage of *Shigella* infected cells by at least 2.5-fold at 6 hpi, compared to a control siRNA (Fig 3B and 3C, S3 Table). Interestingly, despite the comparable increase in the percentage of infected cells, knockdown of these transcripts did not increase *Shigella* intracellular load as efficiently as miR-29b-2-5p (Fig 3C, S3 Table), suggesting that the phenotype induced by the miRNA on infection ensues from its cumulative effect on multiple targets, presumably acting at different stages of infection.

### UNC5C is an important miR-29b-2-5p direct target that modulates *Shigella* binding to host cells

**Fig 4:**
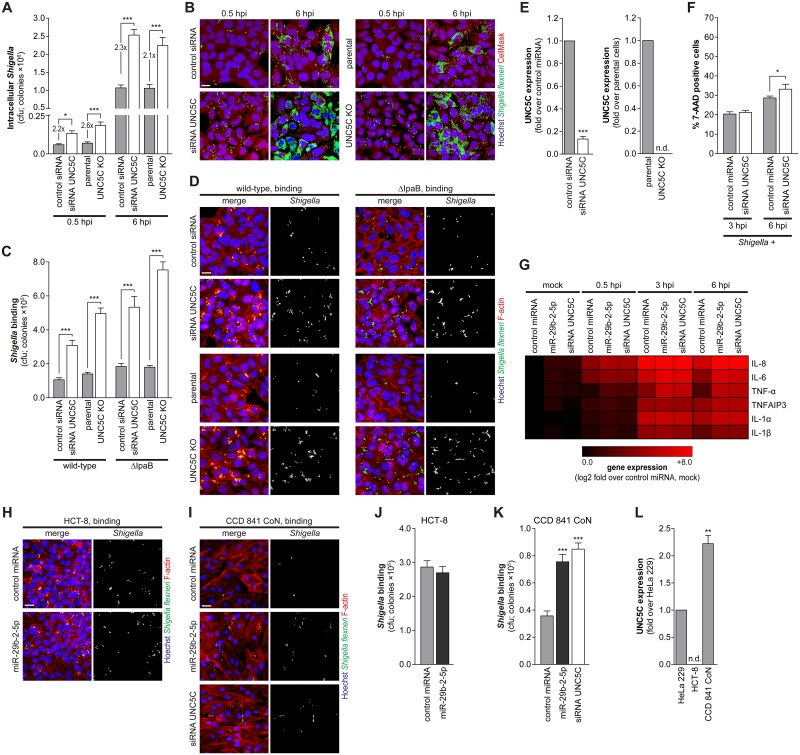
UNC5C is a negative regulator of *Shigella* binding to host cells. A and B. Cfu quantification (A) and representative images (B) of intracellular bacteria in HeLa cells transfected with UNC5C siRNA or control siRNA, as well as UNC5C knockout (UNC5C KO) and parental cells, infected with *Shigella* WT and analyzed at 0.5 and 6 hpi. Scale bar, 20 μm. C and D. Cfu quantification (C) and representative images (D) of bacteria bound to HeLa cells transfected with UNC5C or control siRNA, as well as UNC5C KO and parental cells, incubated with *Shigella* WT or ΔIpaB mutant strain for 10 min. Scale bar, 20 μm. E. UNC5C expression in HeLa cells treated with UNC5C siRNA (left panel) and in parental and UNC5C KO cells (right panel). Results are shown normalized to cells transfected with control siRNA or parental cells, respectively. F. Percentage of 7-AAD positive cells following transfection with UNC5C siRNA or control siRNA for the cell population with internalized *Shigella* (*Shigella* +), analyzed at 3 and 6 hpi. Y-axis was left unchanged to facilitate comparison with Fig 1G. G. Heat-map representing the gene expression analysis of a panel of pro-inflammatory cytokines and downstream genes in mock-treated and *Shigella* infected cells, transfected with control or miR-29b-2-5p miRNA mimics or UNC5C siRNA, analyzed at 0.5, 3 and 6 hpi. Results are shown normalized to mock-treated cells, transfected with control miRNA. H-K. Representative images (H, I) and cfu quantification (J, K) of *Shigella* bound to HCT-8 colon cancer cells (H, J) or CCD 841 CoN normal colon cells (I, K) transfected with miR-29b-2-5p, UNC5C siRNA or control miRNA mimics, and infected with *Shigella* WT. Scale bar, 20 μm. L. UNC5C expression in HeLa, HCT-8 and CCD 841 CoN cells. Results are shown normalized to UNC5C expression levels in HeLa cells. *Shigella* infection was performed at MOI 50 (HeLa and CCD 841 CoN cells) or MOI 10 (HCT-8 cells) for the binding experiments and MOI 10 for intracellular bacterial load (0.5, 3 and 6 hpi). Results are shown as mean ± s.e.m. from 3 (panel E), 4 (panels L and G), 5 (panels A, C, J and K), or 11 (panel F) independent experiments; *P<0.05, **P<0.01, ***P<0.001.

Among the putative miR-29b-2-5p targets with a marked effect on *Shigella* infection, we focused our attention on the top hit of the siRNA screening—UNC5C, a member of the UNC5 netrin-1 receptors. This receptor family has been implicated in various cellular processes [30], including neuronal migration, embryonic angiogenesis and control of cell survival, but has not been previously associated to host-pathogen interactions. Consistent with UNC5C being a target of miR-29b-2-5p, we confirmed that the expression of UNC5C was increased at late times of *Shigella* infection, inversely correlating with miR-29b-2-5p (compare Figs 3D and 2B) and that UNC5C expression was decreased upon transfection of miR-29b-2-5p (Fig 4E). To provide evidence of direct binding of miR-29b-2-5p to UNC5C transcript, we first generated a reporter construct containing the full-length UNC5C 3’UTR cloned downstream of the Renilla luciferase coding sequence (psiCHECK-2-UNC5C 3’UTR). Firefly luciferase expressed from the same vector was used for normalization. As shown in Fig 3F and 3G, miR-29b-2-5p repressed Renilla luciferase activity significantly, in comparison with the control miRNA, denoting direct targeting of the UNC5C 3’UTR by miR-29b-2-5p. Given the length of the UNC5C 3’UTR (>6,700 nt) and the presence of multiple predicted miR-29b-2-5p binding sites (using TargetScan [15] and RNAhybrid [31]), we subsequently generated reporter constructs containing different fragments of the UNC5C 3’UTR, as well as mutant constructs to finely map the miR-29b-2-5p binding sites. This strategy identified of two binding sites in the 5’-end of the UNC5C 3’UTR (Fig 3F and 3G).

Both the knockdown and knockout of UNC5C significantly increased *Shigella* infection, when compared with control siRNA or parental cells, respectively (Figs 4A, 4B and S6A). A comparable increase of infection was observed at 0.5 hpi upon transfection of siRNAs targeting UNC5C, in UNC5C KO cells and upon transfection of miR-29b-2-5p mimic (ca. 2.5-fold; compare Figs 4A, 4B, S6A, 1B, 1C and S1C). At late times post-infection (6 hpi), miR-29b-2-5p treatment led to an increase of *Shigella* intracellular load significantly stronger than that obtained upon UNC5C knockdown or knockout (ca. 2.2- vs 9.9-fold; compare Figs 4A, 4B, S6A, 1B, 1C and S1C), whereas the number of infected cells remained comparable (Figs 1B and 4B). Taken together, these observations support the involvement of UNC5C in the early interaction between *Shigella* and host cells. Accordingly, binding of *Shigella* wild-type, as well as ΔIpaB strain, to host cells was significantly enhanced by UNC5C depletion or knockout when compared with control siRNA transfected or parental cells, respectively (Figs 4C, 4D and S6B). The comparable number of bacteria present within infected cells at intermediate and late stages of infection (3 and 6 hpi) between UNC5C knockdown cells and control cells (S6C Fig) further demonstrates that UNC5C does not affect *Shigella* replication; this is in contrast with the strong increase in *Shigella* intracellular load in cells treated with miR-29b-2-5p (S6C Fig). Moreover, these results further demonstrate that increased invasion cannot per se explain the striking increase in bacterial load observed in cells treated with miR-29b-2-5p at late time points post-infection. Of note, knockdown of UNC5C by RNAi led to a decrease in UNC5C expression similar to that achieved by miR-29b-2-5p mimic (siRNA UNC5C; Fig 4E), and the UNC5C knockout cell line generated through CRISPR/Cas9 mediated genome editing [29] showed a complete absence of UNC5C expression (UNC5C KO; Fig 4E).

We observed a slight increase in cell death upon UNC5C knockdown compared to cells transfected with a control siRNA, at late times post-infection (6 hpi; Figs 4F and S6D). Despite this increase, cell death remained significantly lower than that observed in cells transfected with miR-29b-2-5p (compare Figs 4F, S6D, 1G and S2E). This is most likely related to the fact that miR-29b-2-5p dramatically increases *Shigella* intracellular replication, whereas UNC5C knockdown/knockout enhances *Shigella* binding/invasion to host cells but does not affect intracellular replication. These results indicate that the role of UNC5C in the control of cell survival is not preponderant to its function during *Shigella* infection. We have also confirmed that similarly to miR-29b-2-5p, UNC5C knockdown does not affect *Shigella* intercellular spreading (S3D and S3E Fig). In addition, expression analysis of a panel of pro-inflammatory cytokines and downstream proteins (IL-8, IL-6, TNF-α, TNFAIP3, IL-1α and IL-1β) revealed that miR-29b-2-5p and UNC5C knockdown either did not affect the expression of these genes or slightly increased their expression in mock-treated cells (Figs 4G and S6I). Their expression was sustained or increased in infected cells transfected with miR-29b-2-5p mimic or UNC5C siRNA compared to control cells, at both 3 and 6 hpi (Figs 4G and S6I). These results show that miR-29b-2-5p and UNC5C knockdown are not dampening the pro-inflammatory response to *Shigella* infection.

Consistent with previous reports showing that UNC5C is not expressed in colon cancer cell lines and patient samples due to promoter methylation [32, 33], transfection of miR-29b-2-5p mimic in HCT-8 colon cancer cells did not affect *Shigella* binding to host cells (Figs 4H, 4J and S6E). Of note, we confirmed the lack of UNC5C expression in HCT-8 cells (Fig 4L). A slight, but significant, reduction in *Shigella* binding to cells overexpressing GFP-UNC5C compared to control cells transfected with GFP alone was observed (1.3-fold; S6G Fig). This mild effect is most likely explained by the low transfection efficiency of HCT-8 cells (15–20% for GFP-UNC5C). In agreement, microscopy analysis revealed a significant reduction in *Shigella* bound to cells expressing GFP-UNC5C compared to GFP expressing cells (S6H Fig). Importantly, in CCD 841 CoN cells, a human normal colon cell line that expresses UNC5C at levels significantly higher than HeLa cells (Fig 4L), treatment with miR-29b-2-5p mimic or UNC5C knockdown resulted in an increase of *Shigella* binding to host cells (Figs 4I, 4K and S6F).

Overall, these results demonstrate that the modulation of UNC5C is determinant to the effect of miR-29b-2-5p on *Shigella* binding to host cells, including in colon epithelial cells, the biological target of *Shigella* infection.

### MiR-29b-2-5p, through targeting of UNC5C, positively regulates filopodia formation and *Shigella* capture

**Fig 5:**
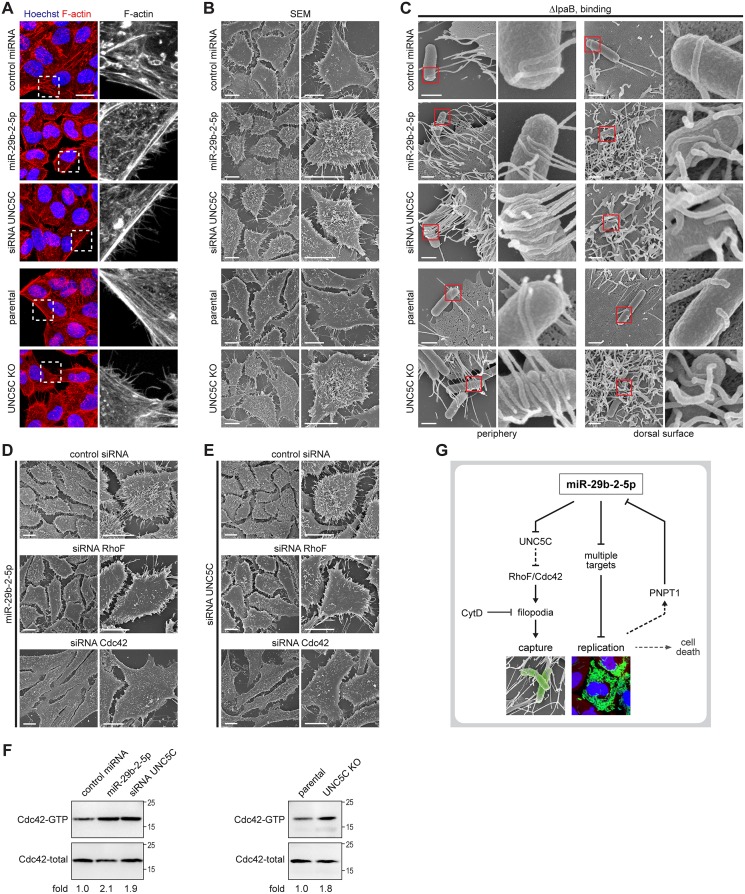
MiR-29b-2-5p, through repression of UNC5C expression, increases filopodia formation and *Shigella* capture by host cells. A. Representative images of HeLa cells treated with UNC5C siRNA, miR-29b-2-5p or control miRNA mimics, as well as UNC5C KO and parental cells, stained for F-actin with fluorescently labeled phalloidin. F-actin staining corresponding to the dashed boxes are enlarged in the rightmost panels. Scale bar, 20 μm. B. Scanning electron microscopy (SEM) of HeLa cells transfected with UNC5C siRNA, miR-29b-2-5p or control miRNA mimics, as well as UNC5C KO and parental cells. Images obtained at lower (left) and higher (right) magnifications are shown for each treatment. Scale bar, 10 μm. C. Scanning electron microscopy of HeLa cells treated with UNC5C siRNA, miR-29b-2-5p or control miRNA mimics, as well as UNC5C KO and parental cells, infected with *Shigella* ΔIpaB mutant strain (MOI 50) for 10 min. Images representative of filopodia at cell periphery (left) or at the dorsal cell surface (right) are shown; red boxes are enlarged in the rightmost panels. Scale bar, 1 μm. D and E. Scanning electron microscopy of HeLa cells co-transfected with miR-29b-2-5p mimic (D) or UNC5C siRNA (E) and control, RhoF or Cdc42 siRNAs. Images obtained at lower (left) and higher (right) magnifications are shown for each treatment. Scale bar, 10 μm. Representative images of the scanning electron microscopy were selected from 3 independent experiments. F. Cdc42 activation in miR-29b-2-5p treated cells, UNC5C knockdown (left panel) and UNC5C knockout cells (right panel), when compared to control. G. Model of the dual role of host miRNA miR-29b-2-5p during *Shigella* infection. MiR-29b-2-5p induces formation of filopodia, through repression of UNC5C expression, thus increasing *Shigella* capture by host cells; in addition, miR-29b-2-5p increases *Shigella* intracellular replication, presumably through repression of several target mRNAs. Internalization and replication of *Shigella* within host cells lead to decreased levels of miR-29b-2-5p, which contributes to a balanced intracellular replication, avoiding premature cell death and favoring the efficient dissemination of *Shigella* to neighboring cells.

Detailed examination of cell morphology, following F-actin staining, revealed that cells transfected with the miR-29b-2-5p mimic or UNC5C siRNA, as well as UNC5C KO cells, display a remarkable increase in the number of cell extensions when compared with control cells (Fig 5A). It has recently been shown that, during the early steps of interaction between *Shigella* and host cells, bacteria establish contacts with pre-existing filopodia extensions on the cell surface [3]. We thus hypothesized that miR-29b-2-5p, through the repression of its target UNC5C, could be increasing *Shigella* interaction with host cells through induction of filopodia formation.

To address this hypothesis, we performed scanning electron microscopy (SEM) experiments, which confirmed a dramatic increase of filopodia, both in periphery and in the dorsal surface of HeLa and CCD 841 CoN colon cells transfected with miR-29b-2-5p mimic or UNC5C siRNA, as well as in UNC5C KO cells (Figs 5B and S7E). Moreover, we observed an increase of host cells with *Shigella* associated with peripheral filopodia, as well as of cells with *Shigella* entrapped by dorsal filopodia (Fig 5C). Since *Shigella* is rapidly internalized following contact with the host cells, these experiments were performed with the *Shigella* ΔIpaB strain, which is able to bind but does not invade host cells, thus allowing easier analysis of the capture step. When using the wild-type strain, an increase of filopodia emanating from the ruffles formed at *Shigella* invasion sites was also observed in miR-29b-2-5p treated cells and UNC5C knockdown and knockout cells (S7A Fig). In agreement with the pivotal role of actin in filopodia dynamics [34, 35], treatment of cells with the inhibitor of actin polymerization cytochalasin D reverted the potentiating effects of miR-29b-2-5p mimic and UNC5C knockdown/knockout on *Shigella* interaction with host cells (S7B–S7D Fig).

Analysis of the effect of UNC5C knockdown on infection by other bacterial pathogens revealed a slight increase of *Salmonella* binding to cells treated with UNC5C siRNA (ca. 1.3-fold, S8A–S8C Fig), which was reflected in a comparable increase of intracellular bacteria at 4 and 20 hpi (S8D–S8F Fig). For *Listeria monocytogenes*, a food-borne gram-positive pathogen [36], a mild increase of binding to UNC5C knockdown cells was also detected (ca. 1.3-fold, S8G–S8I Fig), with a stronger increase detected at 20 hpi (2.4-fold, S8J–S8L Fig). Despite these slight changes, the UNC5C knockdown and consequent filopodia formation did not affect *Salmonella* and *Listeria* binding and invasion of host cells to the same extent as *Shigella*. Concordantly, scanning electron microscopy showed that the degree of interaction of *Salmonella* with the filopodia present in host cells is significantly lower than that observed for *Shigella* infection (compare Fig 5C and S7F Fig). Specifically, we did not detect long-distance *Salmonella* interactions with the longer cell periphery filopodia, as observed for *Shigella*. We did detect interaction of *Salmonella* with shorter dorsal surface filopodia, however this contact was not noticeably increased in cells treated with miR-29b-2-5p mimic or UNC5C siRNA (S7F Fig), as observed for *Shigella* (Fig 5C).

GTPases of the Rho family are small proteins involved in regulating the organization of the actin cytoskeleton, and thereby cell shape, adhesion and movement. Both RhoF (aka Rif) and Cdc42 have been shown to induce filopodia, albeit via distinct mechanisms and with different characteristics [37–39]. Interestingly, the knockdown of either RhoF or Cdc42 abolished the potentiating effect of miR-29b-2-5p mimic and UNC5C knockdown on filopodia formation (Figs 5D, 5E and S9A–S9C) and consequently on *Shigella* binding to host cells (S9D–S9F Fig). Moreover, increased Cdc42 activation, reflected by more GTP-bound Cdc42, was observed in extracts from UNC5C knockdown and knockout cells as well as of miR-29b-2-5p treated cells, when compared to control (Fig 5F).

These results demonstrate that the major event underlying the effect of miR-29b-2-5p, and consequent repression of its target UNC5C, on *Shigella* early interaction with host cells derives from the increased filopodia formation, which promotes bacterial capture.

## Discussion

MicroRNAs are emerging as essential players in the complex interaction between bacterial pathogens and host cells, participating as part of the host defense mechanisms against pathogens, or exploited by the microorganisms to manipulate host cell pathways to promote invasion, survival, replication and dissemination. The identification and characterization of miRNAs modulating infection by specific bacterial pathogens (e.g. *Salmonella* Typhimurium, *Mycobacterium tuberculosis*) is under way, however for most bacterial pathogens, including *Shigella*, this remains essentially unexplored.

In this work, we focus on miR-29b-2-5p, a miRNA with a seed sequence unique in the human genome, which favors *Shigella* infection very efficiently. Detailed characterization of the effect of this miRNA revealed that it plays a dual regulatory role by modulating two distinct steps of *Shigella* infection (Fig 5G): miR-29b-2-5p promotes bacterial capture by dramatically increasing filopodia formation and it increases *Shigella* intracellular replication leading to increased bacterial loads. Interestingly, this miRNA had a mild effect on *Salmonella* binding to host cells and no effect on intracellular replication. Indeed, we show that *Salmonella* binding to host cells is not markedly affected by the increase of filopodia elicited by miR-29b-2-5p. Moreover, we speculate that the differential effect of this miRNA on *Shigella* and *Salmonella* intracellular replication results from their different intracellular lifestyles: it is likely that miR-29b-2-5p is affecting a pathway(s) relevant for *Shigella* replication in the host cytoplasm that is not relevant for *Salmonella*, which mostly replicates in a vacuole.

Interestingly, we observed that miR-29b-2-5p levels are decreased at late times of *Shigella* infection. Regulation of mature miR-29b-2-5p occurs independently of the primary miRNA transcript (pri-miR-29b-2/c) and of the three other mature miRNAs that result from its processing (miR-29b-3p, miR-29c-5p and miR-29c-3p), the levels of which remain unchanged. Importantly, we show that this regulation is dependent on PNPT1, a human polynucleotide phosphorylase with 3’-to-5’ exoribonuclease activity [40], which has been implicated in the degradation of several mature miRNAs (e.g. miR-221 and miR-222) in melanoma cells [41]. To our knowledge, this is the first report of the involvement of an exoribonuclease in the degradation of mature miRNAs during infection by a bacterial pathogen. Moreover, knockdown of PNPT1 led to increased *Shigella* infection both at early and late time points, revealing its relevance in the context of infection, at least in part dependent on miR-29b-2-5p regulation. Future work will focus on characterizing how PNPT1 activity is modulated during *Shigella* infection that, as demonstrated in this study, does not occur at the transcriptional level and is not related to protein steady state levels. Our data showing that increased *Shigella* intracellular replication sustained by high levels of miR-29b-2-5p induce host cell death, suggest that dampening of miR-29b-2-5p levels can constitute a bacterial strategy to favor balanced intracellular replication, thus avoiding premature host cell death and allowing bacteria spreading to neighboring cells. Alternatively, it is conceivable that regulation of miR-29b-2-5p levels constitutes part of the host response, in an attempt to counteract *Shigella* infection. Importantly, the relatively high basal levels of miR-29b-2-5p in epithelial cells at the start of the infection, and the fact that reduction of miR-29b-2-5p levels occurs only at later stages of infection, ensure efficient capture of *Shigella* by host cell filopodia and consequent invasion.

A major challenge in the analysis of miRNA function concerns the identification of the key miRNA targets underlying a given phenotype [13]. Here, we have used a combination of transcriptome analysis followed by a targeted RNAi screening approach to pinpoint, among the putative targets of miR-29b-2-5p, those relevant in the context of *Shigella* infection. Treatment with siRNAs targeting 6 of these genes (UNC5C, ZCCHC24, MAFB, SH3RF2, VAV3 and PALLD) significantly increased the percentage of infected cells. In this manuscript we focused on the role of UNC5C, although the other hits clearly deserve further analysis. VAV3 is a guanine nucleotide exchange factor preferentially for RhoG, RhoA, and to a lesser extent Rac1 [42], which has been involved in *Shigella* invasion. Surprisingly, VAV3 depletion increases *Shigella* infection, and therefore it is likely that this effect is unrelated to invasion. Another interesting candidate is PALLD, a protein involved in regulation of actin dynamics, namely binding, bundling and polymerization [43]. MAFB is a transcription factor with important functions in development and differentiation, which has been shown to act as regulator of type I interferon transcription and of actin organization in macrophages [44, 45]. SH3RF2 and ZCCHC24 are significantly less studied and currently there is no relevant information that may relate their function to *Shigella* infection. Importantly, knockdown of none of the 46 tested genes increased *Shigella* intracellular load to the levels achieved by miR-29b-2-5p. Given that miR-29b-2-5p acts in two distinct steps of the bacterial infection cycle, it is likely that multiple independent targets are responsible for the two phenotypes. Accordingly, we found that UNC5C is the miRNA direct target responsible for increased bacterial capture by host cells, and that its knockdown fully recapitulates the miRNA phenotype observed at early stages of *Shigella* infection. Moreover, we could uncouple the effect of miR-29b-2-5p on bacterial binding to host cells from its effect on intracellular bacterial replication, by generating a UNC5C knockout cell line. This cell line constitutes an interesting resource to study not only the early stages of *Shigella*-host cell interaction, but also the intracellular steps of *Shigella* infection, overcoming current limitations associated with the low adhesion activity of *Shigella* in vitro and the use of bacterial strains expressing ectopic adhesins (e.g. *E*. *coli* AfaE adhesin [46]) or coated with poly-lysine [47].

Interestingly, we demonstrate that miR-29b-2-5p and its target UNC5C are involved in the regulation of filopodia formation in epithelial cells. Consistent with our results showing that UNC5C negatively regulates filopodia formation in human cells, the ortholog of UNC5C in *C*. *elegans*, UNC5, has been shown to inhibit filopodia protrusion in neuronal growth cones [48, 49]. Filopodia are thin actin rich cell protrusions, which extend beyond the cell body, and are present in multiple cell types. By sensing mechanical and chemical cues in the extracellular milieu, filopodia are involved in several processes, including cell motility, cell-cell communication, cell-matrix adhesion and exploration of the environment [50, 51]. Viruses, such as murine leukemia virus, human immunodeficiency virus and human papilloma virus, have been shown to adhere to and glide along the side of filopodia to entry sites located at the cell body [52]. Likewise, several bacteria, including *Yersinia pseudotuberculosis* and *Campylobacter jejuni*, have also been shown to adhere to filopodia prior to internalization [53, 54]. Filopodia have also been shown to play an important role in the capture of *Shigella* [3]. Our results demonstrate that miR-29b-2-5p, through repression of UNC5C, induces filopodia formation, leading to a dramatic increase in *Shigella* capture and consequent bacterial invasion. Interestingly, we observed *Shigella* interaction with filopodia present both in the cell periphery, as well as in the dorsal cell surface: in general, we found bacteria at a distance of the cell surface associated with the longer cell periphery filopodia, whereas bacteria closer to the cell body are mostly associated with dorsal surface structures. Based on these observations, we hypothesize that the cell periphery extensions are involved in long-distance capture of *Shigella*, whereas the shorter dorsal surface filopodia function in maintaining the captured bacteria closer to the cell body, prior to invasion. It has been previously suggested that capture of *Shigella* mediated by filopodia increases the amount of bacteria invading per entry site [3]. Our results showing a similar number of *Shigella* associated per cell, despite the increase in filopodia, rather indicate that filopodia increase the probability of bacterial capture, but not the efficiency of invasion at a given site. Mechanistically, our results demonstrate that the formation of filopodia sustained by miR-29b-2-5p and knockdown/knockout of UNC5C is dependent on Cdc42 and RhoF Rho-GTPases. It has been shown that Cdc42 induced filopodia are generally short and project from the cell periphery, whereas the structures induced by RhoF are usually longer and arise from both the cell periphery and dorsal surface [37–39]. Interestingly, the cells treated with miR-29b-2-5p or knockdown/knockout of UNC5C present a combination of both phenotypes, displaying both short and long filopodia projections.

This study constitutes the first demonstration that host miRNAs modulate infection by *Shigella*. In this context, it is particularly interesting that miR-29b-2-5p acts on two independent steps of the infection process. Further studies will reveal if the concomitant regulation of multiple steps of the interaction between bacterial pathogens and host cells by miRNAs is a recurrent feature, which may explain the very strong phenotypes observed for some miRNAs.

## Materials and methods

### Cell culture

Human epithelial HeLa-229 cells (HeLa CCL-2.1, ATCC) were cultured in DMEM GlutaMAX containing 1.0 g l^**-1**^ glucose (Life Technologies, 21885), human colon cancer HCT-8 cells (ATCC CCL-244) in RPMI 1640 GlutaMAX (Life Technologies, 72400), human colon normal CCD 841 CoN cells (ATCC CRL-1790) in DMEM-F12 GlutaMAX (Life Technologies, 31331), supplemented with 10% fetal bovine serum (Biochrom, S0615-1047D). Cells were maintained at 37°C in a 5% CO_2_ humidified atmosphere.

### Bacterial strains

*Shigella flexneri* serotype 5 strain M90T,*Salmonella* enterica serovar Typhimurium strain SL1344 expressing GFP constitutively from a chromosomal locus [55], and *Listeria monocytogenes* serovar 1/2a EGD-e are referred to as wild-type (WT) in this study. *Shigella flexneri* M90T, its plasmid cured derivative BS176, and *Listeria monocytogenes* serovar 1/2a EGD-e were kindly provided by Prof. J. Vogel (IMIB, University Würzburg, Germany). The isogenic ΔIcsA and ΔIpaB mutants were generated by deleting the coding sequence of IcsA and IpaB, respectively, and inserting the kanamycin cassette via phage lambda Red recombinase-mediated homologous recombination. The following primer pairs were used to generate the deletion mutants: ΔIcsA 5'-TTTTCAGGGGTTTATCAACCACTTACTGATAATATAGTGCGTGTAGGCTGGAGCTGCTTC-3' and 5'-AGAGAAATGCAGGACATCAACACGCCCTGCATTTTTATTAGGTCCATATGAATATCCTCCTTAG-3', ΔIpaB 5'-CTGGTTTTCCTCTTGCCAAAATATTGACTTCCACTGAGCTGTGTAGGCTGGAGCTGCTTC-3' and 5'-GGTATAAGGTCTGTGAGGGTTTTACCTATTATTTTGCCAAGGTCCATATGAATATCCTCCTTAG-3'; the following primers were used to verify the deletion: ΔIcsA 5'-AACACAGCTCTCATGTTTTGG-3' and 5'-AGGCATACCATCATGTGCAC-3', ΔIpaB 5'-AGTGCTTCGAACTCGTAATTC-3' and 5'-TATGCGCTGCAATCTGCTG-3'. *Shigella flexneri* strains expressing GFP and DsRed were obtained by transforming the pXG-1 [56] and pCIG (kindly provided by Dr. A. Zumsteg, MPI-IB, Berlin, Germany) plasmids, respectively.

*Shigella* and *Salmonella* were grown aerobically in Luria broth (LB), and *Listeria* was grown in Brain Heart Infusion (BHI) medium. When appropriate, media were supplemented with antibiotics (*Shigella* GFP: 20 μg ml^-1^ chloramphenicol, *Shigella* DsRed: 20 μg ml^-1^ ampicillin, *Salmonella* GFP: 20 μg ml^-1^ chloramphenicol).

### Bacterial infections

For *Shigella* and *Salmonella* infections, overnight bacterial cultures were diluted 1:100 in LB and grown aerobically at 37°C with shaking until OD_600_ 0.4 (*Shigella*) or OD_600_ 2.0 (*Salmonella*). Bacteria were harvested by centrifugation (12,000 g, 2 min) and resuspended in complete medium. Unless otherwise specified, infections were performed at multiplicity of infection (MOI) of 10 (*Shigella*) or 25 (*Salmonella*). After addition of bacteria, cells were centrifuged at RT at 2,000 g for 15 min (*Shigella*) or 250 g for 10 min (*Salmonella*), followed by incubation at 37°C in a 5% CO_2_ humidified atmosphere for 15 min or 20 min, respectively. The medium was then replaced with fresh medium supplemented with 50 μg ml^-1^ gentamicin (defined as time point 0 of infection) and incubated for 30 min to kill extracellular bacteria. After this incubation, the medium was replaced and cells were maintained in medium supplemented with 10 μg ml^-1^ gentamicin, until analysis. For *Listeria* infections, overnight cultures were diluted 1:50 in BHI medium and grown at 37°C with shaking until OD_600_ 0.7. Dilution of bacterial cultures and infections were performed as described above for *Salmonella*.

For the quantification of *Shigella* spreading, infections were performed as described above; cells were infected at MOI 5 (control miRNA) or 1.25 (miR-29b-2-5p mimic and UNC5C siRNA) to ensure comparable levels of infection between the different treatments at 0.5 hpi.

For treatments with heat-killed (HK) *Shigella*, after reaching OD_600_ 0.4, bacteria were harvested by centrifugation as described above, washed twice in PBS, incubated for 3 h at 100°C and then resuspended in complete medium. Incubation of cells with heat-killed *Shigella* was performed as described above for live bacteria.

For binding experiments, cells were exposed to *Shigella* WT or ΔIpaB mutant strain (MOI 50), centrifuged at 2,000 g for 15 min and incubated for 10 min at 37°C, 5% CO_2_. Cells were then extensively washed with PBS to remove non-bound bacteria and immediately processed for microscopy, cfu assay or RNA extraction. For *Salmonella* and *Listeria* a MOI 50 was used for binding experiments, and cells were processed as described above immediately after the centrifugation step (250 g, 10 min). To block actin polymerization, when indicated HeLa-229 cells were treated with 0.5 μg ml^-1^ cytochalasin D (CytD; Sigma, C8273), or DMSO (control), for 90 min prior to infection.

To quantify intracellular bacteria replication by colony forming units (cfu) assay, at the indicated time points cells were washed three times in PBS and lysed in PBS containing 0.1% Triton-X-100. The cell lysates were then serially diluted in PBS and plated on LB agar plates.

### sRNA and plasmid transfection

Transfection of miRNAs mimics and siRNAs into HeLa-229 and HCT-8 cells was performed with Lipofectamine RNAiMAX (Life Technologies), using a standard reverse transfection protocol at a final miRNA/siRNA concentration of 50 nM, as described previously [20]. Co-transfections experiments were performed with 25 nM of each miRNA/siRNA at a final concentration of 50 nM. For CCD 841 CoN cells, forward transfection at a final miRNA/siRNA concentration of 50 nM using Lipofectamine RNAiMAX was performed, according to the manufacturer’s instructions.

The library of miRNA mimics corresponding to all the human mature miRNAs annotated in miRBase 19 (2,042 mature miRNA sequences) was obtained from Dharmacon, GE Healthcare; for the targeted siRNA screening, selected siRNAs were ‘cherry-picked’ from the human genome-wide siRNA library stock plates (siGENOME SMARTPools, 4 siRNAs per gene, Dharmacon, GE Healthcare); gene IDs and gene symbols are included in S3 Table. MiRIDIAN hsa-miR-29b-2-5p mimic (C-301149-01), siGENOME SMARTpool UNC5C siRNA (M-010628-01), siGENOME SMARTpool Cdc42 siRNA (M-005057-01), siGENOME SMARTpool RhoF siRNA (M-008316-00-01), siGENOME non-targeting siRNA #5 (D-001210-05) and miRIDIAN microRNA mimic negative control #4 (cel-miR-231; CN-004000-01) were purchased from Dharmacon, GE Healthcare. PNPT1 (SASI_Hs01_00228542), RRP41 (SASI_Hs01_00239912) and XRN1 (SASI_Hs01_00212296) pre-designed siRNAs were purchased from Sigma. The hsa-miR-29b-2-5p miRCURY LNA Power inhibitor (4103894–101) and the negative control A miRCURY LNA Power inhibitor (199006–101) were purchased from Exiqon.

The miRNA and siRNA screenings were performed in 384-wellplates, essentially as described previously [20]. Briefly, miRNAs and siRNAs were transferred robotically from stock library plates and arrayed in 384-well plates (Viewplate-384 black, clear bottom, PerkinElmer). Transfection of miRNA mimics and siRNAs was performed using a standard reverse transfection protocol with Lipofectamine RNAiMAX, at a final miRNA/siRNA concentration of 50 nM; 1.5×10^3^ HeLa cells were seeded per well. Forty-eight hours after transfection, cells were infected with *Shigella* (MOI 25), as described above. Cells were fixed at 6 hpi and counterstained with HCS CellMask Deep Red stain (1:15,000; Life Technologies) and Hoechst 33342 (1:5,000; Life Technologies), according to the manufacturer’s instructions. Transfection was optimized using a toxic siRNA targeting ubiquitin C. The screening was performed in triplicate.

For cfu assay, RNA isolation and microscopy, 5.0×10^4^ HeLa and CCD 841 CoN cells or 8.0×10^4^ HCT-8 cells were seeded per well in 24-well plates. Infections with *Shigella* and *Salmonella* were performed as described above, 60 h (*Shigella*) or 48 h (*Salmonella*) after transfection with miRNA mimics or siRNAs.

For HCT-8 cells transfection with plasmid DNA, 7.0×10^4^ cells were seeded per well in 24-well plates, and grown for 48 h to reach ca. 70% confluency. Cells were then transfected with Lipofectamine 3000 (Life Technologies), with 1 μg of pEGFP-C1 or pEGFP-C1-UNC5C, according to the manufacturer’s instructions. Transfected cells were infected as described above, 48 h after plasmid transfection.

### Immunofluorescence, fluorescence microscopy and analysis

Cells were fixed with 4% paraformaldehyde (PFA) for 15 min at RT, permeabilized with 0.5% Triton-X-100 in PBS for 10 min. Blocking was performed for 30 min in 1% Bovine Serum Albumin (BSA) in PBS. Cells were then stained with a primary antibody directed against *Shigella* LPS (1:150, 2 h RT; Gentaur, YCC310-130) or *Listeria* (1:750, 2 h RT; antibodies-online, ABIN 237765) diluted in blocking buffer. Cells were further washed and incubated with the corresponding secondary antibody conjugated with Alexa Fluor 488 (1:400, 1 h RT; Life Technologies, A21441).

For F-actin staining, following the blocking step cells were incubated with Alexa Fluor 594 Phalloidin (1:150, 1 h RT; Life Technologies, A12381).

When indicated, cells were stained with HCS CellMask Deep Red stain (1:10,000, 1 h RT; Life Technologies, H32721) and nuclei were counterstained with Hoechst 33342 (1:5,000, 15 min RT; Life Technologies, 62249). Slides were mounted in Vectashield (VectorLabs).

To confirm the efficacy of the gentamicin treatment, a control experiment based on immunofluorescence labelling of *Shigella* before and after a permeabilization step was performed. Briefly, HeLa-229 cells were infected with a *Shigella* strain constitutively expressing mCherry, as described above. At 0.5 and 6 hours after infection, cells were washed in PBS three times and immediately incubated with a rabbit polyclonal anti-*Shigella* antibody (1:50, 1 h RT; Gentaur, YCC310-130). Cells were then further washed with PBS, fixed with 4% PFA and incubated with Alexa Fluor 488 conjugated anti-rabbit secondary antibody (1:250, 1 h RT; Life Technologies, A21441). Of note, incubation with the primary antibody was performed in live cells, prior to fixation, since fixation can permeabilize the cell membrane. For each time point, a parallel set of samples was fixed and permeabilized prior to incubation with the primary antibody to ensure complete staining of intracellular bacterial, as described above.

For quantification of bacterial infection, image acquisition was performed using an ImageXpress Micro (Molecular Devices) or an Operetta (PerkinElmer) automated high-content screening fluorescence microscope at a 20× magnification; a total of 9–16 images were acquired per coverslip/well, corresponding to approximately 2,500 cells analyzed per experimental condition and replicate. Image analysis was performed using the ‘Multi-Wavelength Cell Scoring’ application module implemented in MetaXpress software (Molecular Devices), or Columbus image analysis software (PerkinElmer). Briefly, nuclei and cells were segmented based on the Hoechst and CellMask stainings, respectively; cells were then classified as positive or negative for *Shigella* depending on the total area of bacterial staining. For the miRNA and siRNA screenings, miRNA mimics or siRNAs that decreased the total number of host cells to less than 65% of the control were excluded from further analysis.

Time-lapse *Shigella* infection experiments were performed in an Operetta automated high-content screening fluorescence microscope equipped with the live cell chamber option for temperature and CO_2_ control. Prior to infection, cells were treated with Hoechst (1:100,000, 1 h RT) and then infected as described above, in 384-well plates. Images were acquired at 15 min intervals from 0.5 to 6 hpi, at 37°C, 5% CO_2_.

Confocal microscopy images were obtained using a laser scanning Leica SP5 confocal microscope (Leica Microsystems). For the analysis of the number of bacteria per cell, at least 50 infected cells per condition and independent experiment were counted manually from maximum-projected Z-stack confocal images.

### Analysis of *Shigella* spreading

For quantification of *Shigella* spreading, image acquisition was performed using an Operetta (PerkinElmer) automated high-content screening fluorescence microscope at a 10× magnification; a single image was acquired per well, corresponding to an area of ca. 1.4 mm^2^, approximately 2,000 cells were analyzed per experimental condition and replicate. Image analysis was performed using Columbus image analysis software (PerkinElmer). Briefly, *Shigella* infection foci were identified based on texture and intensity features, using the ‘Find Texture Regions’ building block implemented in Columbus image analysis software, and the size of each individual foci was determined using the ‘Calculate Morphology Properties’ building block. Infection foci of very small dimension (below 400 μm^2^; average HeLa-229 area 500 μm^2^) and containing low bacterial load were excluded since they represent non-productive infections; infection foci touching the borders of the images were also excluded, to improve the overall accuracy of the measurements. At least 500 individual foci from a total of 5 independent experiments were plotted per experimental treatment. *Shigella* ΔIcsA mutant strain, defective in intercellular spreading was used as a control.

### RNA isolation and quantitative real-time PCR

Total RNA, including the small RNA fraction, was isolated in TRIzol (Life Technologies) and extracted by phenol-chloroform followed by isopropanol precipitation.

For quantification of gene expression, total RNA was reverse-transcribed using hexameric random primers (Life Technologies) and M-MLV reverse transcriptase (Life Technologies), according to the manufacturer’s instructions. Real-time quantitative analysis was performed using SsoAdvanced Universal SYBR Green Supermix (BioRad) according to the manufacturer’s instructions, using a CFX96 Touch Real-Time PCR detection system (BioRad). The following primer pairs were used: GFP 5'-ATGCTTTTCCCGTTATCCGG-3' and 5’-GCGTCTTGTAGTTCCCGTCATC-3'; DsRed 5'-TCATCTACAAGGTGAAGTTC-3' and 5’-AGCCCATAGTCTTCTTCT-3'; UNC5C 5'-GATTGTGATAGCAGTGAT-3' and 5'-CGATGATTCTTCCGATAC-3'; pri-miR-29b-2/c 5'-TTGCCACTTGAGTCTGTT-3' and 5'-CTTCTCTACTGTCACCTCTC-3'; Cdc42 5’-GCCAAGAACAAACAGAAGCCT-3’ and 5’- ACTTGACAGCCTTCAGGTCA-3’; RhoF 5’-AGCAAGGAGGTGACCCTGAAC-3’ and 5’- CCGCAGCCGGTCATAGTC-3’; β-actin 5'-CCTGTACGCCAACACAGTGC-3' and 5'-ATACTCCTGCTTGCTGATCC-3'. Expression was normalized to β-actin.

For miRNA quantification, reverse transcription was performed using the miRCURY LNA Universal cDNA synthesis kit (Exiqon) followed by qRT-PCR using miRCURY LNA SYBR Green master mix (Exiqon) and predesigned mercury LNA PCR primer sets (Exiqon), according to the manufacturer’s instructions. The following primer sets were used: hsa-miR-29b-2-5p (204208), hsa-miR-29b-3p (204679), hsa-miR-29c-5p (204132), hsa-miR-29c-3p (204729) and U6 (203907). MiRNA expression was normalized to U6.

The 2^-ΔΔCt^ method was used to calculate fold changes.

### Flow cytometry and cell sorting

Analysis of cell viability following infection with *Shigella* was performed using 7-Amino-Actinomycin (7-AAD; BD Biosciences, 51-68981E), a viability dye excluded from cells with intact membranes (viable cells), as described previously [20]. Flow cytometry was performed on a BD Accuri C6 flow cytometer (BD Biosciences); a minimum of 10,000 cells was analyzed for each subpopulation (*Shigella* - and *Shigella* +). FlowJo software (Tree Star Inc) was used for data analysis.

Cell sorting to separate the subpopulation of cells infected with *Shigella* (*Shigella* +) from bystander cells (*Shigella* -) was performed as described previously [20]. HeLa-229 cells infected with *Shigella* expressing GFP or DsRed at MOI 10, were collected at 6 hpi and sorted based on the intensity of the GFP or DsRed signal, using a FACSAria III flow cytometer (BD Biosciences). Control, mock-infected cells were processed in parallel; RNA isolated from the sorted cells was processed as described above.

### Scanning electron microscopy

After washing thoroughly with PBS five times, cells were fixed overnight at 4°C in 2.5% glutaraldehyde in 0.1 M cacodylate buffer (pH 7.2). Samples were then washed five times in 0.1 M cacodylate buffer (pH 7.2), dehydrated through a graded series of 30%, 50%, 70%, 90% acetone solution in 10 min incubations, and incubated six times in 100% acetone for 10 min, followed by critical point drying with CO_2_. Dried specimens were sputter coated with gold/palladium. Samples were analyzed with a JEOL JSM-7500F (JEOL GmbH) or a Zeiss Crossbeam 340 (Zeiss) field emission scanning electron microscope operating at 5Kv. Representative images were selected from 3 independent experiments.

### CRISPR/Cas9-mediated genome editing

The UNC5C knockout HeLa-229 cell line (UNC5C KO) and miR-29b-2 knockdown HeLa-229 cell lines (miR-29b-2 KD#1 and miR-29b-2 KD#2) were generated essentially as described previously [29]. Briefly, sgRNA sequences targeting the first exon of UNC5C or the miR-29b-2 precursor miRNA were designed using the online CRISPR Design Tool (http://tools.genome-engineering.org) from F. Zhang laboratory (MIT, USA). Selected sgRNAs were ordered as single strand DNA oligonucleotides (IDT), with the following sequences: UNC5C_sg1 5’-CACCGCACACCTCTGTCTACGATG-3’ and 5’-AAACCATCGTAGACAGAGGTGTGC-3’; UNC5C_sg2 5’-CACCGACAGCGGCCCGCTGCGGACT-3’ and 5’-AAACAGTCCGCAGCGGGCCGCTGTC-3’; miR-29b-2_sg1 5’-CACCGTTCTGGAAGCTGGTTTCACA-3’ and 5’-AAACTGTGAAACCAGCTTCCAGAAC-3’; miR-29b-2_sg2 5’-CACCGAATGGTGCTAGATACAAAGA-3’ and 5’-AAACTCTTTGTATCTAGCACCATTC-3’; miR-29b-2_sg3 5’-CACCGCCTAAAACACTGATTTCAAA-3’ and 5’-AAACTTTGAAATCAGTGTTTTAGGC-3’. The oligos were annealed and cloned directionally into the px330 vector using the BbsI enzyme; the px330 plasmid encodes a chimeric guide RNA and a human codon-optimized *S*. *pyogenes* Cas9 [29]. Equimolar amounts of the constructs encoding the guide RNAs targeting UNC5C or the miR-29b-2 genomic regions were transfected into HeLa-229 cells seeded on 24-well plates, using FuGENE HD (Promega, E2311) or Turbofect (Thermo Scientific, R0531) transfection reagent, according to the manufacturer’s instructions. To isolate clonal populations of edited cells, four days after transfection the cells were plated into 96-well plates, at a limiting dilution. Colonies originated from single cells were expanded and cell fractions were collected for genomic analysis. The genomic region encompassing the target sites of the designed sgRNAs was amplified using the following primers: UNC5C 5’-GTCGTTATTTCTTCGGACTGCTTC-3’ and 5’-AAGGAGGGAAGGAAGAAGCTAAG-3’; miR-29b-2 5’-GTGCTTGTGTCCTGATGAAGTA-3’ and 5’-AAATCGGTCAGCCTGTGTAAG-3’; genome editing was evaluated by Sanger sequencing. Knockout of UNC5C and knockdown of miR-29b-2-5p was validated by qRT-PCR, as described above.

### Dual-luciferase reporter assay

The full length and fragments of the UNC5C 3′UTR were amplified by PCR and cloned into the dual luciferase reporter vector psiCHECK-2 (Promega) using PmeI or XhoI and NotI restriction sites. Mutants of miR-29b-2-5p putative binding sites were generated by site directed mutagenesis. The following oligonucleotides were used for cloning of the UNC5C 3’UTR, fragments and mutagenesis: full-length 5’- CGTGATGTTTAAACCACCATGCTGGAAGGGGAAA-3’ and 5’-ACGAGCGGCCGCTGGCTGACAGAATAAATGCAGG-3’; (1–2372) 5’- CGTGATGTTTAAACCACCATGCTGGAAGGGGAAA-3’ and 5’- GTACGAGCGGCCGCCTCATTGCTACAAACATCCACTG-3’; (2252–4681) 5’- CGTGATGTTTAAACATAAAATCTCTCTCAGCCCACC-3’ and 5’- GTACGAGCGGCCGCTATGAGGCACTCGAGAGGTA-3’; (4556–6730) 5’- CGTGATGTTTAAACTTGTGAAGCAGTACATCATTGC-3’ and 5’- ACGAGCGGCCGCTGGCTGACAGAATAAATGCAGG-3’; (1–612) 5’- CGATCGCTCGAGCCACCATGCTGGAAGGGGAAA-3’ and 5’- GTACGAGCGGCCGCGATTCTCCACTTCCCTGATAC-3’; (580–1680) 5’-CGATCGCTCGAGGTGTTACTGTTTGTATCAGGG-3’ and 5’- GTACGAGCGGCCGCGTCTAGATATCAAGATGCCAAG-3’; (1619–2372) 5’- CGATCGCTCGAGGCCTTCCTGACTGGTTGCAAAT-3’ and 5’- ACGAGCGGCCGCTGGCTGACAGAATAAATGCAGG-3’; mutant site1 5’- AGCTGAGGAGGAAATCCAGAATGCACTTCACAGGCAAGAC-3’ and 5’- GTCTTGCCTGTGAAGTGCATTCTGGATTTCCTCCTCAGCT-3’; mutant site 2 5’- GACTCTAAACTGGAGATCAATTCTCATTGTGGGTCTGTCAC-3’ and 5’- GTGACAGACCCACAATGAGAATTGATCTCCAGTTTAGAGTC-3’.

For luciferase reporter assay, HeLa cells were seeded in 96-well plates (8×10^3^ cells/well) one day before transfection and were transfected with 200 ng of the constructs containing the wild-type or mutated sequences of the 3′UTR-UNC5C using FuGENE HD Transfection reagent. Twenty-four hours after plasmid transfection, cells were transfected with cel-miR-231 or miR-29b-2-5p mimics (final concentration 50 nM) and the experiment was stopped after an additional 48 h. Firefly and Renilla luciferase activities were measured using the Dual-Luciferase Reporter Assay Kit (Promega, E2920) according to the manufacturer’s protocol. Results were expressed as normalized Renilla/Firefly luciferase activity ratios.

### Protein extracts and western-blot

Cells were washed in PBS, collected in Laemmli’s sample buffer and separated in 10% or 12% SDS-PAGE, followed by Western-blotting. The following antibodies were used: β-actin (1:3,000; Sigma, A2228) and PNPT-1 (1:750, Santa Cruz, sc-365049). Anti-mouse secondary antibody coupled to horseradish peroxidase were used (1:10,000; GE Healthcare, NA931). Signals were detected using SuperSignal West Dura Extended Duration Substrate (Pierce, 34075) and an ImageQuant LAS 4000 CCD camera (GE Healthcare).

### Cdc42 pulldown activation assay

Activation of Cdc42 was determined using the Cdc42 Pull-down Activation Assay Biochem Kit (Cytoskeleton, BK034), according to the manufacturer’s instructions. Briefly, prior to analysis HeLa cells were transfected in a 6-well plate (10 wells per condition, 1.5×10^5^ cells/well) using a standard reverse transfection protocol with cel-miR-231, miR-29b-2-5p mimic or UNC5C siRNA, at a final concentration of 50 nM, and incubated during 72 h. For UNC5C KO and parental cells, cells were grown for 48 h (10 wells per condition, 2.4×10^5^ cells/well). All cells were serum-starved for 24 h before collection. After starvation, cells were washed with PBS and scraped in lysis buffer. Equal amounts of total protein (600 μg) of each lysate were then incubated with the PAK-PBD beads, according to the manufacturer’s instructions. Total and activated GTP-bound Cdc42 (pulldown) were analyzed by SDS-PAGE and Western-blot as described above. Relative Cdc42 activation was calculated by densitometric analysis of the intensities of the Cdc42 bands from the pulldown (GTP-Cdc42) and total Cdc42 present in the sample, which was used for normalization.

### RNA sequencing and computational RNA-seq analysis

Library preparation and deep-sequencing was performed by Vertis Biotechnology AG, as described previously [20]. RNA-seq analysis was performed using the READemption pipeline version 0.3.0 [57], with Segemehl version 0.1.7 [58]. Reads were mapped against the human (build GRCh37) and *Shigella flexneri* (NCBI Reference Sequence NC_008258) genomes. Analysis of differential gene expression was performed with DESeq 1.18.0 [59]. The demultiplexed FASTQ files and gene-wise quantifications have been deposited in NCBI’s Gene Expression Omnibus and are accessible through GEO Series accession numbers GSE75746 and GSE75747.

### Statistical analysis

No statistical methods were used to predetermine sample size. The investigators were not blinded to allocation during experiments and outcome assessment. The data are presented as mean ± standard error of the mean (s.e.m.), of at least three independent experiments. The exact number of experiments performed for each panel is indicated in Figure legends. Statistical analysis was performed using Prism Software (GraphPad). For statistical comparison of datasets from two conditions, two-tailed Student’s t-test was used; for data from three or more conditions, ANOVA with Tukey’s or Dunnet’s post-hoc test was used. A P-value of 0.05 or lower was considered significant.

## Supporting information

S1 Fig Top-10 miRNAs increasing *Shigella* infection identified through a high-throughput screening of a genome-wide library of miRNA mimics. A and B. Percentage (A) and representative images (B) of *Shigella* infected cells following treatment with the 10 highest ranking miRNAs increasing bacterial infection, identified through the microscopy-based high-throughput screening. Cells treated with control miRNA are shown for comparison. Scale bar, 100 μm. C. Quantification of *Shigella* by qRT-PCR in HeLa cells infected with *Shigella* WT, upon treatment with miR-29b-2-5p or control miRNA mimics, and analyzed at three times post-infection (0.5, 3 and 6 hpi). D. Immunofluorescence labelling of *Shigella* before and after a permeabilization step, performed at 0.5 and 6 hpi. Scale bar, 20 μm.*Shigella* infection was performed with MOI 10. Results are shown as mean ± s.e.m. from 3 (panel A) or 8 (panel C) independent experiments, normalized to control miRNA; *P<0.05, **P<0.01, ***P<0.001.
(TIF)

S2 Fig MiR-29b-2-5p increases *Shigella* infection. A. Fluorescence microscopy images extracted from the time-lapse microscopy analysis of HeLa cells infected with *Shigella* WT, upon treatment with miR-29b-2-5p or control miRNA mimics; Images corresponding to 1, 2, 3, 4, 5 and 6 hpi are shown; dashed boxes are shown enlarged below the corresponding images. Full time-lapse sequence is included as supplementary material (S1 Video). Scale bar, 100 μm. B. Quantification of *Shigella* by qRT-PCR in HeLa cells transfected with miR-29b-2-5p or control miRNA mimics, and incubated with *Shigella* WT or ΔIpaB mutant strain for 10 min. C. Cfu quantification of intracellular *Shigella* in HeLa cells infected with various *Shigella* MOIs (10, 50 and 100) and analyzed at 0.5, 3 and 6hpi. Y-axis was left unchanged to facilitate comparison with Fig 1C. D. Cfu quantification of intracellular *Shigella* in HeLa cells at 3 and 6 hpi, upon treatment with miR-29b-2-5p or control miRNA mimics. Results are normalized to bacteria internalized at 0.5 hpi, to discriminate effects at late time post-infection. E. Percentage of 7-AAD positive cells following treatment with control or miR-29b-2-5p miRNA mimics for mock treated cells, total cells and *Shigella* - cell population, analyzed at 3 and 6 hpi. *Shigella* infection was performed at MOI 50 for binding and MOI 10 for intracellular replication (0.5, 3 and 6 hpi) experiments. Results are shown as mean ± s.e.m. from 5 (panels B, C and D) or 15 (panel E) independent experiments, normalized to control miRNA; *P<0.05, **P<0.01, ***P<0.001.
(TIF)

S3 Fig MiR-29b-2-5p does not affect *Salmonella* infection or *Shigella* intercellular spreading. A-C. Representative images (A), cfu quantification of intracellular bacteria (B) and *Shigella* quantification by qRT-PCR (C) of HeLa cells infected with *Shigella* ΔIcsA mutant deficient in spreading (MOI 100), upon treatment with miR-29b-2-5p or control miRNA mimics, and analyzed at 3 hpi. D and E. Representative images with corresponding image segmentation (D) and quantification of infection foci area (E) of HeLa cells infected with wild-type *Shigella* upon treatment with miR-29b-2-5p mimics, UNC5C siRNA or control miRNA mimics, and analyzed at 3 hpi. *Shigella* ΔIcsA mutant is shown for comparison. Infection foci marked in red (panel D) touch the border of the image, and were excluded from analysis. F-H. Representative images (F), cfu quantification of intracellular bacteria (G) and *Salmonella* quantification by qRT-PCR (H) of HeLa cells infected with *Salmonella* WT (MOI 25), upon treatment with miR-29b-2-5p or control miRNA mimics, and analyzed at two times post-infection corresponding to early and late times of *Salmonella* infection (4 and 20 hpi). I-K. Representative images (I), cfu quantification (J) and quantification by qRT-PCR (K) of *Salmonella* bound to HeLa cells transfected with miR-29b-2-5p or control miRNA mimics and incubated with *Salmonella* WT or Δ4 mutant strain for 15 min. For A, F and I, scale bar, 20 μm; for D, 100 μm. Results are shown as mean ± s.e.m. from 5 independent experiments, normalized to control miRNA; *P<0.05, **P<0.01,***P<0.001.
(TIF)

S4 Fig Knockdown of the exonuclease PNPT1 increases *Shigella* infection. A-C. Representative images (A), cfu quantification (B) and quantification by qRT-PCR (C) of *Shigella* bound to HeLa cells transfected with PNPT1 or control siRNA. Scale bar, 20 μm. D-F. Representative images (D), cfu quantification of intracellular bacteria (E) and *Shigella* quantification by qRT-PCR (F) of HeLa cells infected with *Shigella*, upon transfection with PNPT1 and control siRNA. Scale bar, 20 μm. For panel F, results are shown normalized to control siRNA at 0.5 hpi. G. PNPT1 expression, quantified by qRT-PCR, in the total cell population, *Shigella* + and *Shigella* - fractions, at 0.5, 3 and 6 hpi. HeLa cells were infected with *Shigella* WT expressing GFP at MOI 10 and subjected to cell sorting to separate the population of cells with internalized bacteria (*Shigella* +) and bystander cells (*Shigella* -). H. PNPT1 protein levels in mock treated and *Shigella* infected (MOI 100) HeLa cells, determined at 0.5, 3 and 6 hpi. I. PNPT1 expression, quantified by qRT-PCR, in the total cell population, *Shigella* + and *Shigella* - fractions, at 6 hpi. Samples were prepared as in panel G. J. PNPT1 expression, determined by Western-blot, in HeLa cells treated with PNPT1 or control siRNA. *Shigella* infection was performed at MOI 50 for binding and MOI 10 for intracellular bacterial load (0.5 and 6 hpi). Results are shown as mean ± s.e.m. from 4 (panel G) or 5 (panels B, C, E and F) independent experiments; *P<0.05, **P<0.01, ***P<0.001.
(TIF)

S5 Fig Inhibition of miR-29b-2-5p expression decreases *Shigella* infection. A. MiR-29b-2-5p expression levels in HeLa cells treated with control or miR-29b-2-5p miRNA inhibitor (left panel) and in parental and miR-29b-2 knockdown cells generated by CRISPR/Cas9 genome editing (miR-29b-2 KD#1 and #2; right panel). Results are shown normalized to cells transfected with control inhibitor or parental cells, respectively. B-D. Representative images (B), cfu quantification (C) and quantification by qRT-PCR (D) of *Shigella* bound to HeLa cells transfected with control or miR-29b-2-5p inhibitor, as well as miR-29b-2 knockdown and parental cells. Scale bar, 20 μm. E-G. Representative images (E), cfu quantification (F) and quantification by qRT-PCR (G) of intracellular bacteria in HeLa cells transfected with control or miR-29b-2-5p inhibitor, as well as miR-29b-2 knockdown and parental cells, infected with *Shigella* WT and analyzed at 0.5 and 6 hpi.. Scale bar, 20 μm. *Shigella* infection was performed at MOI 50 for binding and MOI 10 for intracellular bacterial load (0.5 and 6 hpi). Results are shown as mean ± s.e.m. from 5 independent experiments; **P<0.01, ***P<0.001.
(TIF)

S6 Fig UNC5C knockdown or knockout increases *Shigella* binding to host cells, but not intracellular replication. A. Quantification of *Shigella* by qRT-PCR in HeLa cells transfected with UNC5C siRNA or control siRNA, as well as UNC5C knockout (UNC5C KO) and parental cells, infected with *Shigella* WT and analyzed at 0.5 and 6 hpi. B. Quantification of *Shigella* by qRT-PCR in HeLa cells transfected with UNC5C or control siRNA, as well as UNC5C KO and parental cells, incubated with *Shigella* WT or ΔIpaB mutant strain for 10 min. C. Distribution of the number of *Shigella* per infected cell at different times post-infection (binding, 0.5, 3 and 6 hpi), in HeLa cells transfected with UNC5C siRNA, miR-29b-2-5p or control miRNA mimics. Results are shown for at least 50 infected cells, per condition and independent experiment. Values in the X-axis correspond to the extremities of the defined bins. D. Percentage of 7-AAD positive cells following treatment with control or UNC5C siRNA for mock treated cells, total cells and *Shigella* - cell population, analyzed at 3 and 6 hpi. Y-axis was left unchanged to facilitate comparison with S2E Fig. E and F. Quantification by qRT-PCR of *Shigella* bound to HCT-8 colon cancer cells (E) or CCD 841 CoN normal colon cells (F) transfected with miR-29b-2-5p, UNC5C siRNA or control miRNA mimics. G and H. Cfu quantification (G) and representative images (H) of *Shigella* bound to HCT-8 cells overexpressing GFP-UNC5C or GFP alone. Scale bar, 20 μm. I. Expression analysis of a panel of pro-inflammatory cytokines and downstream genes in mock-treated and *Shigella* infected cells, transfected with control miRNA, miR-29b-2-5p or UNC5C siRNA, analyzed at 0.5, 3 and 6 hpi. Results are shown normalized to mock-treated cells, transfected with control miRNA. P value is shown compared to control miRNA at the corresponding time point. *Shigella* infection was performed at MOI 50 (HeLa and CCD 841 CoN cells) or MOI 10 (HCT-8 cells) for binding and MOI 10 for intracellular bacterial load (0.5 and 6 hpi). Results are shown as mean ± s.e.m. from 4 (panel C and I), 5 (panels A, B, E and F), 7 (panel G) or 11 (panel D) independent experiments; *P<0.05, **P<0.01, ***P<0.001.
(TIF)

S7 Fig Inhibition of filopodia dynamics abrogates the effect of miR-29b-2-5p and UNC5C on the interaction of *Shigella* with host cells. A. Scanning electron microscopy of HeLa cells treated with UNC5C siRNA, miR-29b-2-5p or control miRNA mimics, as well as UNC5C KO and parental cells, infected with *Shigella* WT (MOI 50) for 10 min. Scale bar, 1 μm. B-D. Representative images (B), cfu quantification (C) and *Shigella* quantification by qRT-PCR (D) of HeLa cells transfected with UNC5C siRNA, miR-29b-2-5p or control miRNA mimics, as well as UNC5C KO and parental cells. Cells were pre-treated with DMSO or cytochalasin D, followed by incubation with *Shigella* WT (MOI 50) for 10 min. Scale bar, 20 μm. E. Scanning electron microscopy of CCD 841 CoN normal colon cells treated with UNC5C siRNA, miR-29b-2-5p or control miRNA mimics. Images obtained at lower (left) and higher (right) magnifications are shown for each treatment. Scale bar, 10 μm. F. Scanning electron microscopy of HeLa cells treated with UNC5C siRNA, miR-29b-2-5p or control miRNA mimics, infected with *Salmonella* Δ4 mutant strain (MOI 50) for 15 min. Scale bar, 1 μm. Representative images of the scanning electron microscopy were selected from 3 independent experiments. Results are shown as mean ± s.e.m. from 4 (panel D) and 5 (panel C) independent experiments, normalized to control miRNA or parental cells; ***P<0.001.
(TIF)

S8 Fig Impact of UNC5C knockdown on *Salmonella* and *Listeria* binding to host cells is less pronounced than that on *Shigella*. A-F. Representative images (A, D), cfu quantification (B, E) and quantification by qRT-PCR (C, F) of *Salmonella* WT interaction with HeLa cells transfected with UNC5C or control siRNA, at binding (A-C), or 4 and 20 hpi (D-F). G-L. Representative images (G, J), cfu quantification (H, K) and *Listeria* quantification by qRT-PCR (I, L) of HeLa cells infected with *Listeria* WT, upon transfection with UNC5C or control siRNA, and analyzed at three times post-infection: binding (G-I) or 4 and 20 hpi (J-L)). *Salmonella* and *Listeria* infection were performed at MOI 50 for binding and MOI 25 for intracellular bacterial load (4 and 20 hpi). For A, D and G, scale bar, 20 μm; for J, 50 μm. Results are shown as mean ± s.e.m. from 5 independent experiments; *P<0.05, **P<0.01, ***P<0.001.
(TIF)

S9 Fig Enhancement of filopodia formation mediated by miR-29b-2-5p is dependent on the RhoF and Cdc42 Rho-GTPases. A. Scanning electron microscopy of HeLa cells co-transfected with control miRNA and control, RhoF or Cdc42 siRNAs. Images obtained at lower (left) and higher (right) magnifications are shown for each treatment. Scale bar, 10 μm. Representative images of the scanning electron microscopy were selected from 3 independent experiments. B and C. RhoF (B) and Cdc42 (C) expression in HeLa cells transfected with RhoF, Cdc42 and control siRNAs. Results are shown normalized to cells transfected with control siRNA. D–F. Representative images (D), cfu quantification (E) and qRT-PCR quantification (F) of *Shigella* binding to HeLa cells co-transfected with RhoF, Cdc42 or control siRNAs and UNC5C siRNA, miR-29b-2-5p or control miRNA mimics. Scale bar, 20 μm. Results are shown as mean ± s.e.m. from 5 independent experiments, normalized to control miRNA (panel F) or control siRNA (panels B and C); ***P<0.001.
(TIF)

S1 Table Comparison of the top 10 miRNAs increasing *Shigella* infection and their effect on *Salmonella* infection. Results of the percentage of *Shigella* infected cells for the top 10 miRNAs increasing infection identified in the microscopy-based high-throughput screening of a genome-wide library of miRNA mimics. Results of a *Salmonella* screening, previously published by our group (Maudet et al. 2014), are shown for comparison. Of note, the mature miR-365a-3p and miR-365b-3p have the same sequence; in miRBase 13, this miRNA was named miR-365 (result is shown in italic). Results for percentage of infected cells are shown normalized to control miRNA.
(PDF)

S2 Table Identification of miR-29b-2-5p targets relevant for *Shigella* infection. List of 52 genes that are both repressed by miR-29b-2-5p (≥ 1.5-fold down-regulation; reads ≥ 25 for cells transfected with control miRNA) and have increased expression in *Shigella* infected cells (6 hpi; ≥2-fold up-regulation *Shigella* + population relative to mock; ≤ 2-fold *change Shigella* - population relative to mock; reads ≥25 for mock).
(PDF)

S3 Table Results of the targeted RNAi screening to identify putative miR-29b-2-5p targets relevant for *Shigella* infection. Percentage of HeLa cells infected with *Shigella* WT upon treatment siRNAs targeting 46 genes. SiRNAs that decreased cell viability to less than 65% of control were excluded from further analysis and are highlighted in gray. Results for percentage of infected cells and cell viability are shown normalized to control siRNA.
(PDF)

S1 Video Time-lapse microscopy of *Shigella* infection in HeLa cells transfected with control miRNA or miR-29b-2-5p mimics. Images extracted at different time points are shown in S2A Fig.
(AVI)
