# Supplementary figures and images for: Analysis of host microRNA function uncovers a role for miR-29b-2-5p in Shigella capture by filopodia
Source: PLoS Pathog. 2017 Apr 10;13(4):e1006327. doi: 10.1371/journal.ppat.1006327 (PMC5398735; doi:10.1371/journal.ppat.1006327)

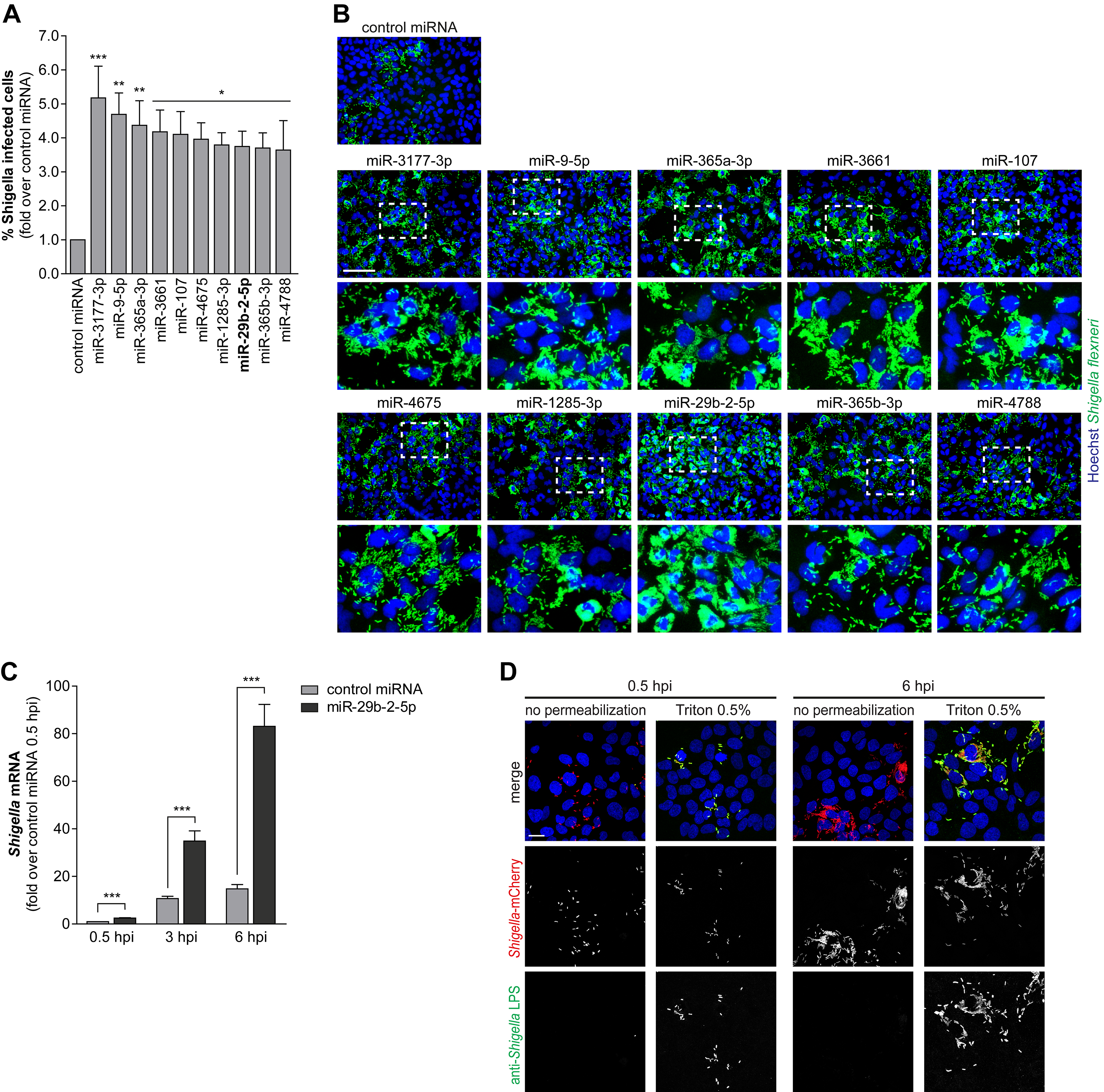

Supplement: S1 Fig — A and B. Percentage (A) and representative images (B) of Shigella infected cells following treatment with the 10 highest ranking miRNAs increasing bacterial infection, identified through the microscopy-based high-throughput screening. Cells treated with control miRNA are shown for comparison. Scale bar, 100 μm. C. Quantification of Shigella by qRT-PCR in HeLa cells infected with Shigella WT, upon treatment with miR-29b-2-5p or control miRNA mimics, and analyzed at three times post-infection (0.5, 3 and 6 hpi). D. Immunofluorescence labelling of Shigella before and after a permeabilization step, performed at 0.5 and 6 hpi. Scale bar, 20 μm.Shigella infection was performed with MOI 10. Results are shown as mean ± s.e.m. from 3 (panel A) or 8 (panel C) independent experiments, normalized to control miRNA; *P<0.05, **P<0.01, ***P<0.001. (TIF) [file ppat.1006327.s001.tif]

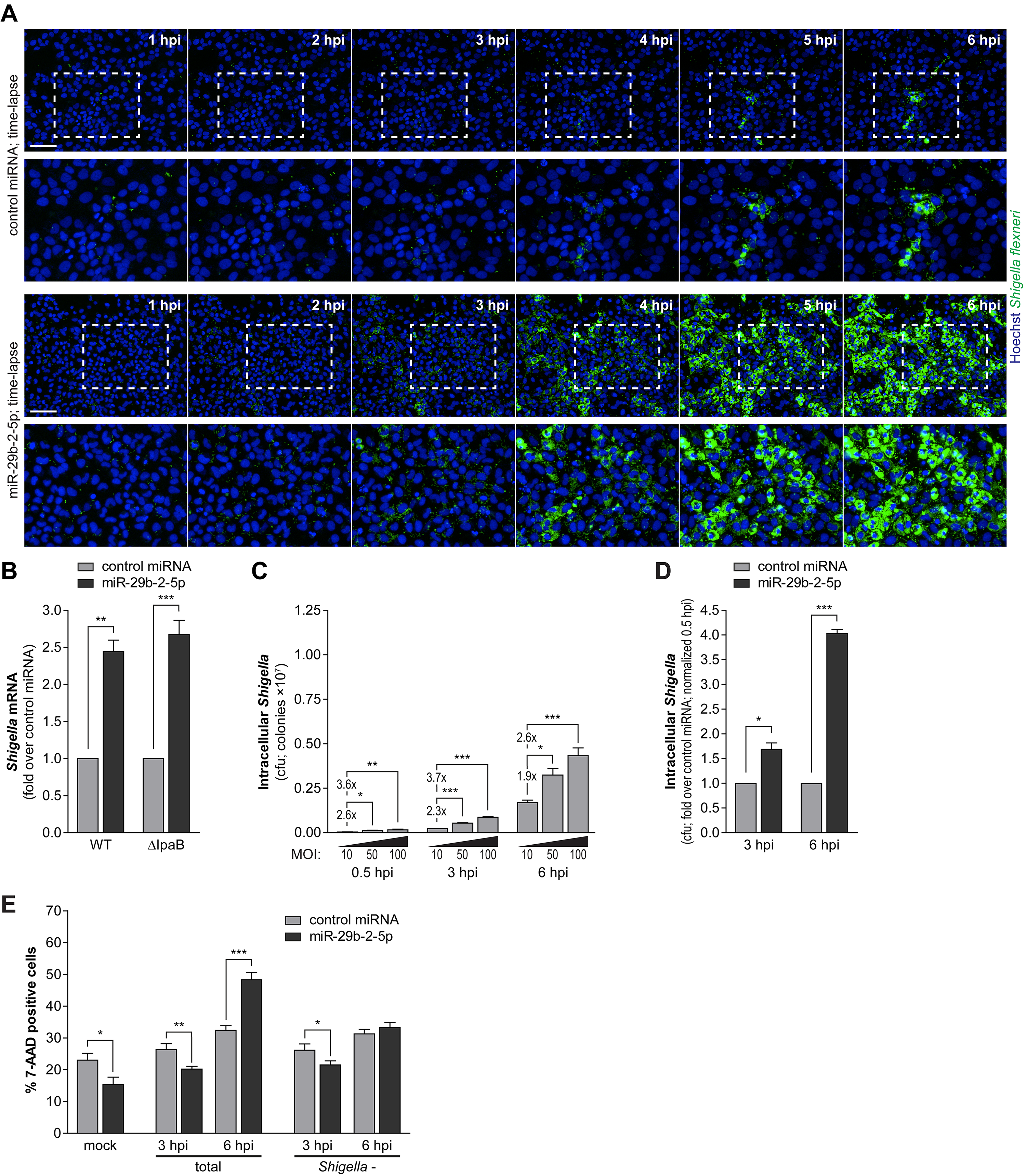

Supplement: S2 Fig — A. Fluorescence microscopy images extracted from the time-lapse microscopy analysis of HeLa cells infected with Shigella WT, upon treatment with miR-29b-2-5p or control miRNA mimics; Images corresponding to 1, 2, 3, 4, 5 and 6 hpi are shown; dashed boxes are shown enlarged below the corresponding images. Full time-lapse sequence is included as supplementary material (S1 Video). Scale bar, 100 μm. B. Quantification of Shigella by qRT-PCR in HeLa cells transfected with miR-29b-2-5p or control miRNA mimics, and incubated with Shigella WT or ΔIpaB mutant strain for 10 min. C. Cfu quantification of intracellular Shigella in HeLa cells infected with various Shigella MOIs (10, 50 and 100) and analyzed at 0.5, 3 and 6hpi. Y-axis was left unchanged to facilitate comparison with Fig 1C. D. Cfu quantification of intracellular Shigella in HeLa cells at 3 and 6 hpi, upon treatment with miR-29b-2-5p or control miRNA mimics. Results are normalized to bacteria internalized at 0.5 hpi, to discriminate effects at late time post-infection. E. Percentage of 7-AAD positive cells following treatment with control or miR-29b-2-5p miRNA mimics for mock treated cells, total cells and Shigella - cell population, analyzed at 3 and 6 hpi. Shigella infection was performed at MOI 50 for binding and MOI 10 for intracellular replication (0.5, 3 and 6 hpi) experiments. Results are shown as mean ± s.e.m. from 5 (panels B, C and D) or 15 (panel E) independent experiments, normalized to control miRNA; *P<0.05, **P<0.01, ***P<0.001. (TIF) [file ppat.1006327.s002.tif]

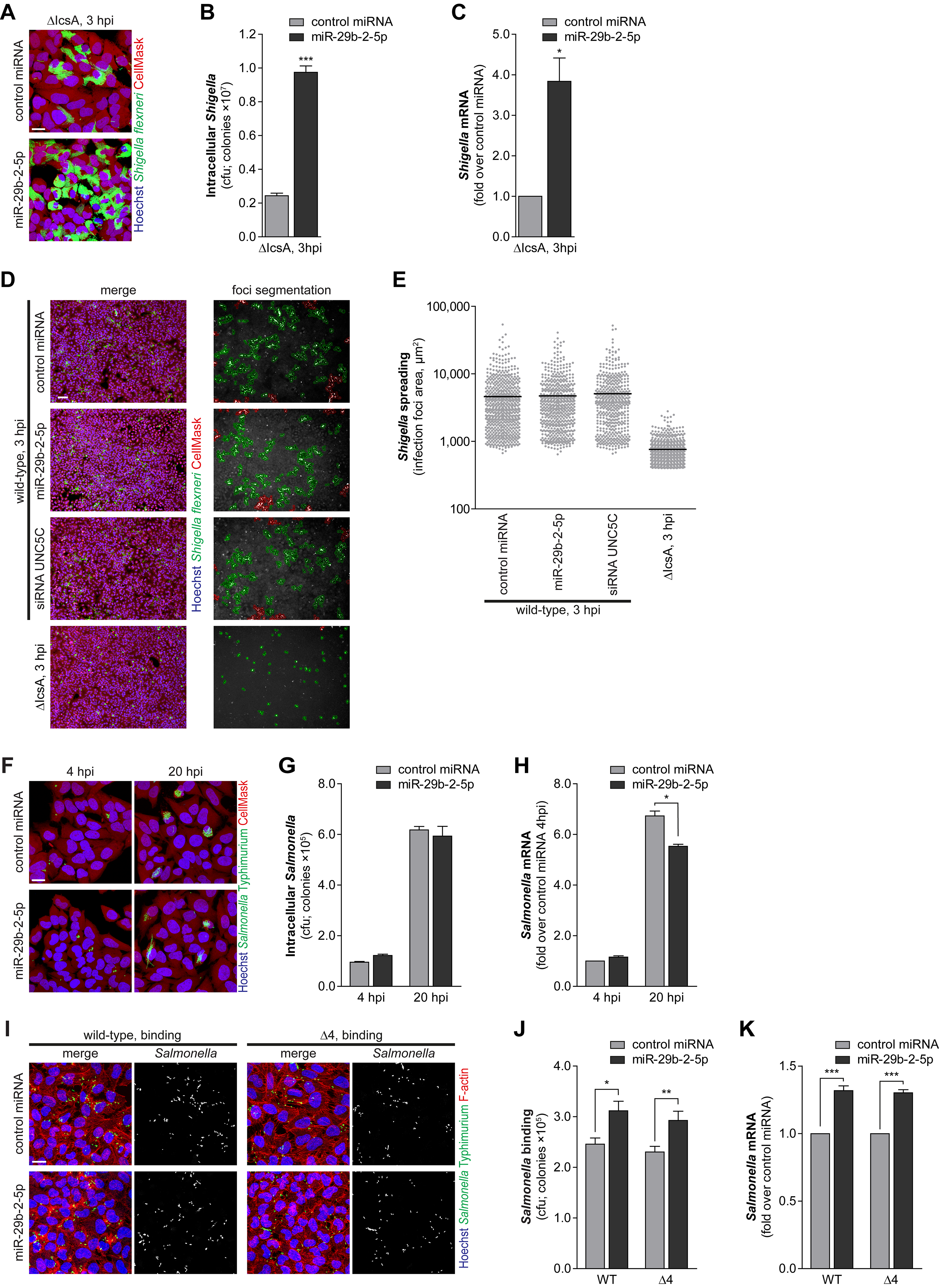

Supplement: S3 Fig — A-C. Representative images (A), cfu quantification of intracellular bacteria (B) and Shigella quantification by qRT-PCR (C) of HeLa cells infected with Shigella ΔIcsA mutant deficient in spreading (MOI 100), upon treatment with miR-29b-2-5p or control miRNA mimics, and analyzed at 3 hpi. D and E. Representative images with corresponding image segmentation (D) and quantification of infection foci area (E) of HeLa cells infected with wild-type Shigella upon treatment with miR-29b-2-5p mimics, UNC5C siRNA or control miRNA mimics, and analyzed at 3 hpi. Shigella ΔIcsA mutant is shown for comparison. Infection foci marked in red (panel D) touch the border of the image, and were excluded from analysis. F-H. Representative images (F), cfu quantification of intracellular bacteria (G) and Salmonella quantification by qRT-PCR (H) of HeLa cells infected with Salmonella WT (MOI 25), upon treatment with miR-29b-2-5p or control miRNA mimics, and analyzed at two times post-infection corresponding to early and late times of Salmonella infection (4 and 20 hpi). I-K. Representative images (I), cfu quantification (J) and quantification by qRT-PCR (K) of Salmonella bound to HeLa cells transfected with miR-29b-2-5p or control miRNA mimics and incubated with Salmonella WT or Δ4 mutant strain for 15 min. For A, F and I, scale bar, 20 μm; for D, 100 μm. Results are shown as mean ± s.e.m. from 5 independent experiments, normalized to control miRNA; *P<0.05, **P<0.01,***P<0.001. (TIF) [file ppat.1006327.s003.tif]

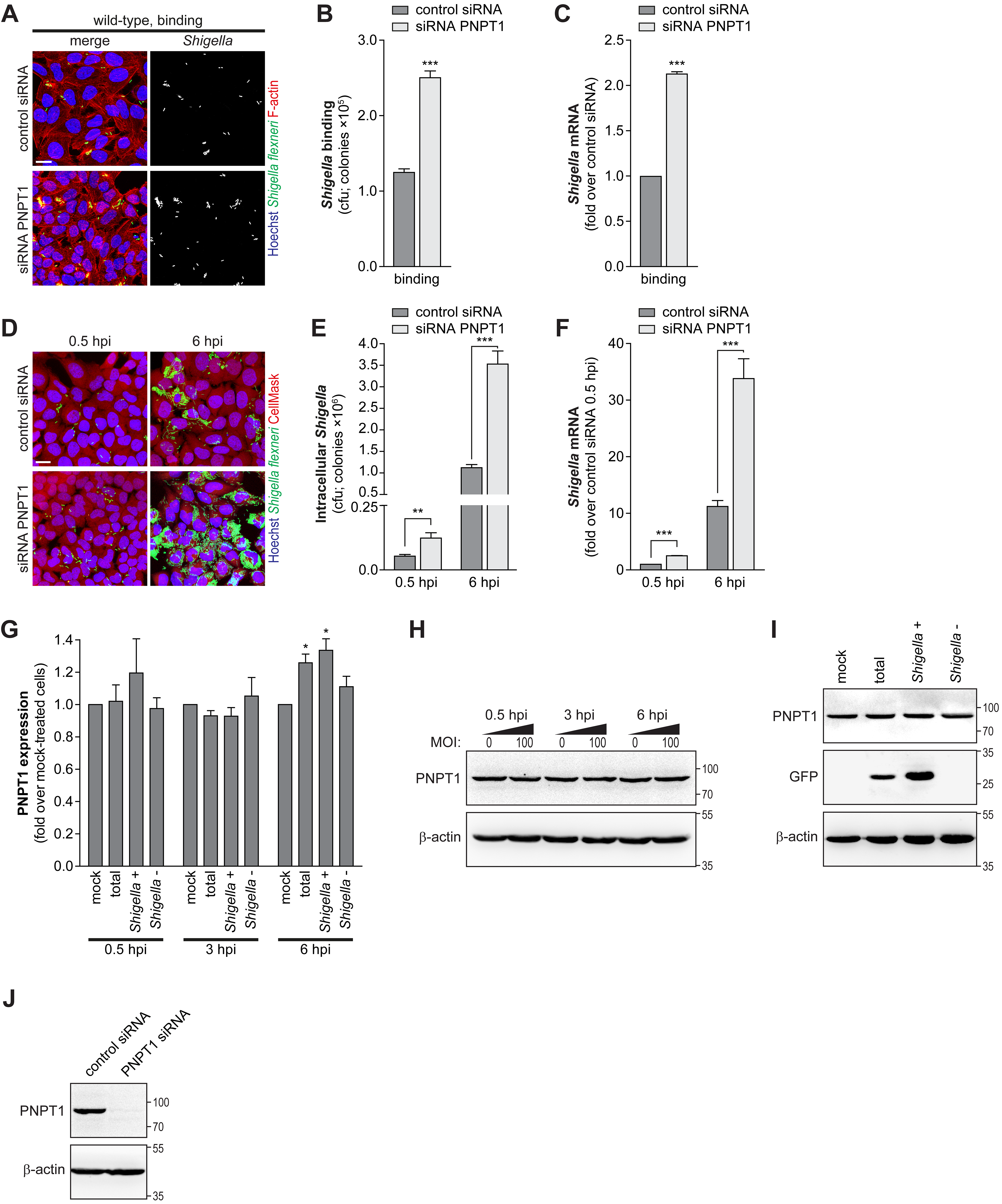

Supplement: S4 Fig — A-C. Representative images (A), cfu quantification (B) and quantification by qRT-PCR (C) of Shigella bound to HeLa cells transfected with PNPT1 or control siRNA. Scale bar, 20 μm. D-F. Representative images (D), cfu quantification of intracellular bacteria (E) and Shigella quantification by qRT-PCR (F) of HeLa cells infected with Shigella, upon transfection with PNPT1 and control siRNA. Scale bar, 20 μm. For panel F, results are shown normalized to control siRNA at 0.5 hpi. G. PNPT1 expression, quantified by qRT-PCR, in the total cell population, Shigella + and Shigella - fractions, at 0.5, 3 and 6 hpi. HeLa cells were infected with Shigella WT expressing GFP at MOI 10 and subjected to cell sorting to separate the population of cells with internalized bacteria (Shigella +) and bystander cells (Shigella -). H. PNPT1 protein levels in mock treated and Shigella infected (MOI 100) HeLa cells, determined at 0.5, 3 and 6 hpi. I. PNPT1 expression, quantified by qRT-PCR, in the total cell population, Shigella + and Shigella - fractions, at 6 hpi. Samples were prepared as in panel G. J. PNPT1 expression, determined by Western-blot, in HeLa cells treated with PNPT1 or control siRNA. Shigella infection was performed at MOI 50 for binding and MOI 10 for intracellular bacterial load (0.5 and 6 hpi). Results are shown as mean ± s.e.m. from 4 (panel G) or 5 (panels B, C, E and F) independent experiments; *P<0.05, **P<0.01, ***P<0.001. (TIF) [file ppat.1006327.s004.tif]

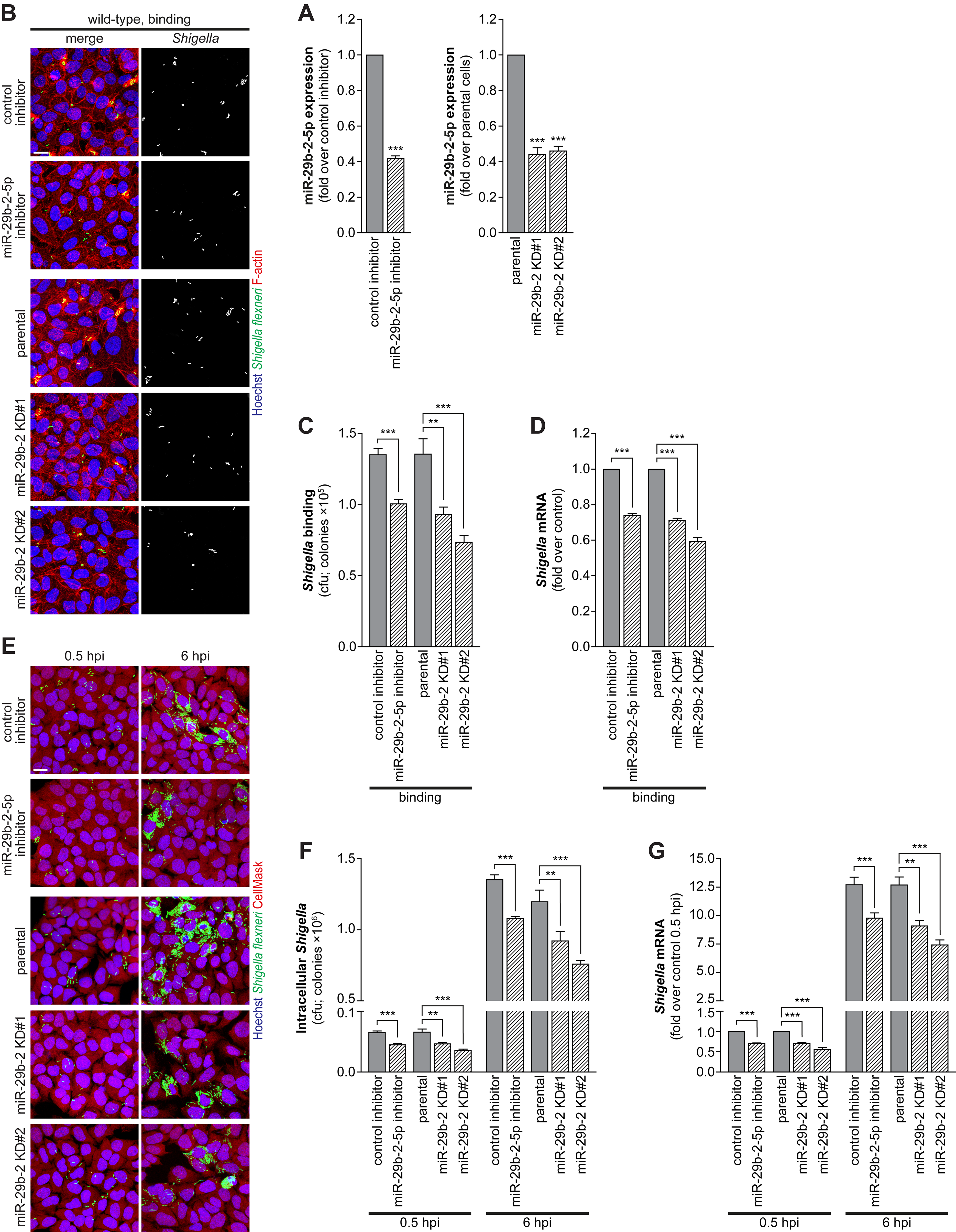

Supplement: S5 Fig — A. MiR-29b-2-5p expression levels in HeLa cells treated with control or miR-29b-2-5p miRNA inhibitor (left panel) and in parental and miR-29b-2 knockdown cells generated by CRISPR/Cas9 genome editing (miR-29b-2 KD#1 and #2; right panel). Results are shown normalized to cells transfected with control inhibitor or parental cells, respectively. B-D. Representative images (B), cfu quantification (C) and quantification by qRT-PCR (D) of Shigella bound to HeLa cells transfected with control or miR-29b-2-5p inhibitor, as well as miR-29b-2 knockdown and parental cells. Scale bar, 20 μm. E-G. Representative images (E), cfu quantification (F) and quantification by qRT-PCR (G) of intracellular bacteria in HeLa cells transfected with control or miR-29b-2-5p inhibitor, as well as miR-29b-2 knockdown and parental cells, infected with Shigella WT and analyzed at 0.5 and 6 hpi.. Scale bar, 20 μm. Shigella infection was performed at MOI 50 for binding and MOI 10 for intracellular bacterial load (0.5 and 6 hpi). Results are shown as mean ± s.e.m. from 5 independent experiments; **P<0.01, ***P<0.001. (TIF) [file ppat.1006327.s005.tif]

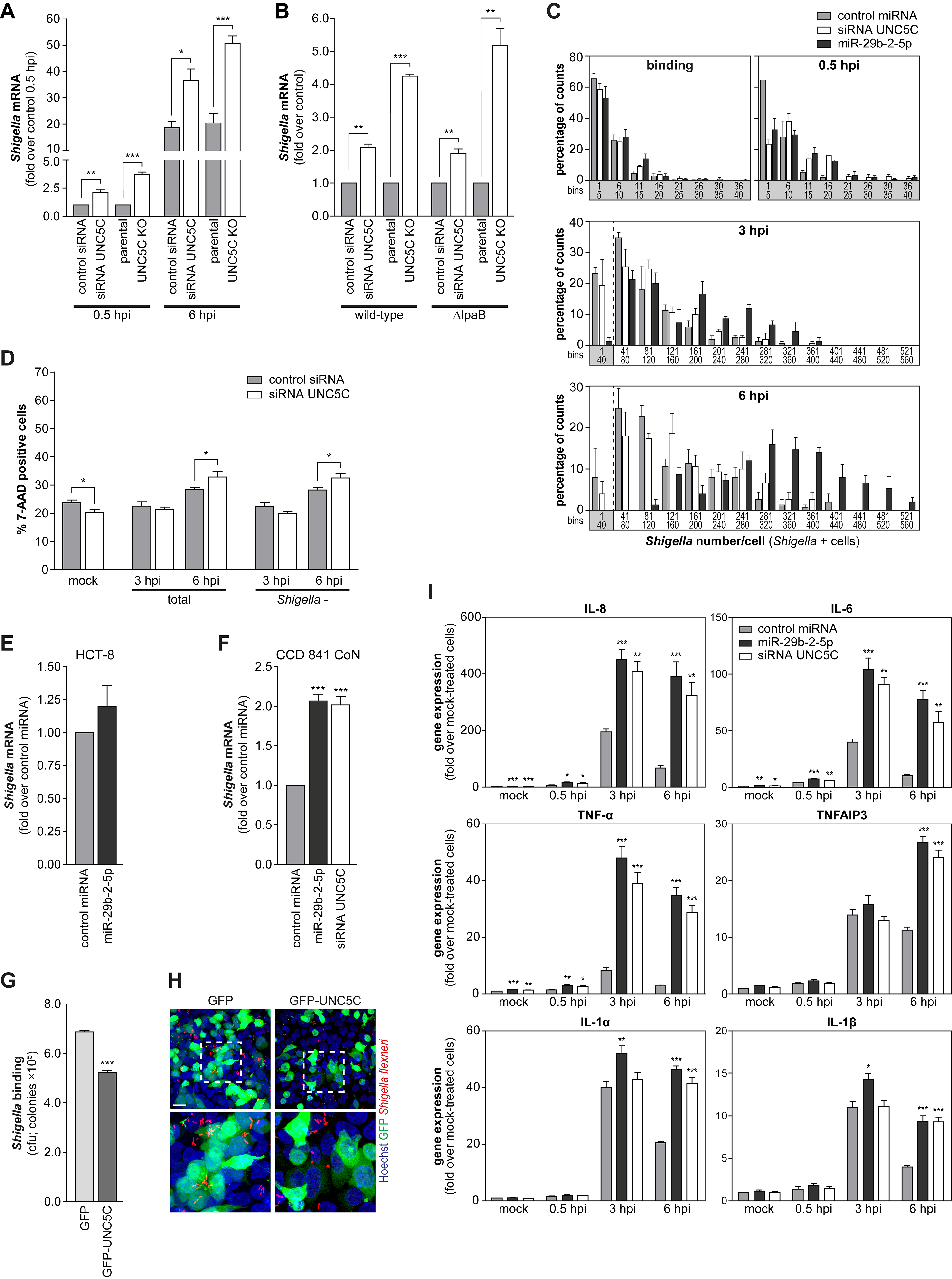

Supplement: S6 Fig — A. Quantification of Shigella by qRT-PCR in HeLa cells transfected with UNC5C siRNA or control siRNA, as well as UNC5C knockout (UNC5C KO) and parental cells, infected with Shigella WT and analyzed at 0.5 and 6 hpi. B. Quantification of Shigella by qRT-PCR in HeLa cells transfected with UNC5C or control siRNA, as well as UNC5C KO and parental cells, incubated with Shigella WT or ΔIpaB mutant strain for 10 min. C. Distribution of the number of Shigella per infected cell at different times post-infection (binding, 0.5, 3 and 6 hpi), in HeLa cells transfected with UNC5C siRNA, miR-29b-2-5p or control miRNA mimics. Results are shown for at least 50 infected cells, per condition and independent experiment. Values in the X-axis correspond to the extremities of the defined bins. D. Percentage of 7-AAD positive cells following treatment with control or UNC5C siRNA for mock treated cells, total cells and Shigella - cell population, analyzed at 3 and 6 hpi. Y-axis was left unchanged to facilitate comparison with S2E Fig. E and F. Quantification by qRT-PCR of Shigella bound to HCT-8 colon cancer cells (E) or CCD 841 CoN normal colon cells (F) transfected with miR-29b-2-5p, UNC5C siRNA or control miRNA mimics. G and H. Cfu quantification (G) and representative images (H) of Shigella bound to HCT-8 cells overexpressing GFP-UNC5C or GFP alone. Scale bar, 20 μm. I. Expression analysis of a panel of pro-inflammatory cytokines and downstream genes in mock-treated and Shigella infected cells, transfected with control miRNA, miR-29b-2-5p or UNC5C siRNA, analyzed at 0.5, 3 and 6 hpi. Results are shown normalized to mock-treated cells, transfected with control miRNA. P value is shown compared to control miRNA at the corresponding time point. Shigella infection was performed at MOI 50 (HeLa and CCD 841 CoN cells) or MOI 10 (HCT-8 cells) for binding and MOI 10 for intracellular bacterial load (0.5 and 6 hpi). Results are shown as mean ± s.e.m. from 4 (panel C and I), 5 (panels A, B, E and [file ppat.1006327.s006.tif]

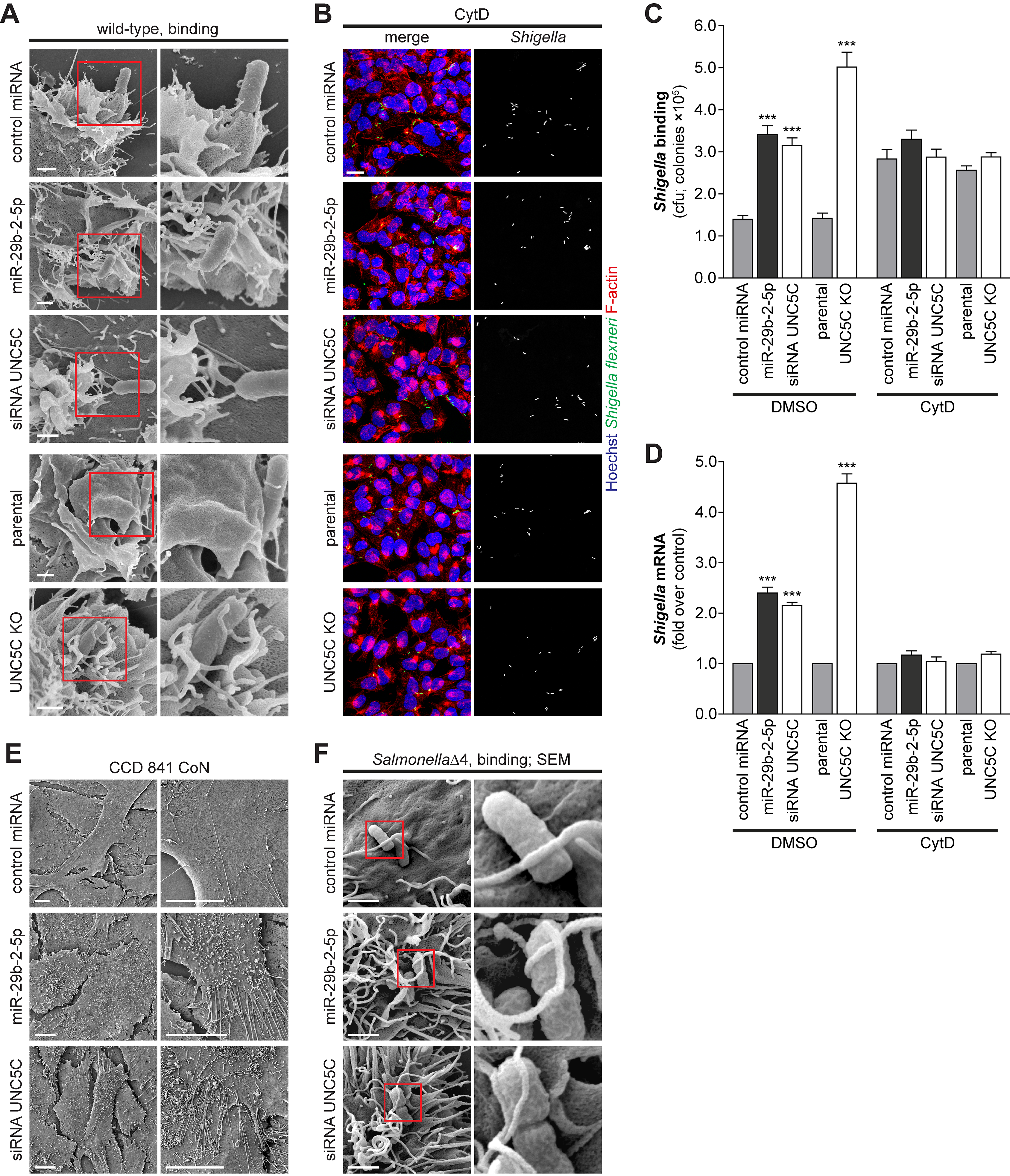

Supplement: S7 Fig — A. Scanning electron microscopy of HeLa cells treated with UNC5C siRNA, miR-29b-2-5p or control miRNA mimics, as well as UNC5C KO and parental cells, infected with Shigella WT (MOI 50) for 10 min. Scale bar, 1 μm. B-D. Representative images (B), cfu quantification (C) and Shigella quantification by qRT-PCR (D) of HeLa cells transfected with UNC5C siRNA, miR-29b-2-5p or control miRNA mimics, as well as UNC5C KO and parental cells. Cells were pre-treated with DMSO or cytochalasin D, followed by incubation with Shigella WT (MOI 50) for 10 min. Scale bar, 20 μm. E. Scanning electron microscopy of CCD 841 CoN normal colon cells treated with UNC5C siRNA, miR-29b-2-5p or control miRNA mimics. Images obtained at lower (left) and higher (right) magnifications are shown for each treatment. Scale bar, 10 μm. F. Scanning electron microscopy of HeLa cells treated with UNC5C siRNA, miR-29b-2-5p or control miRNA mimics, infected with Salmonella Δ4 mutant strain (MOI 50) for 15 min. Scale bar, 1 μm. Representative images of the scanning electron microscopy were selected from 3 independent experiments. Results are shown as mean ± s.e.m. from 4 (panel D) and 5 (panel C) independent experiments, normalized to control miRNA or parental cells; ***P<0.001. (TIF) [file ppat.1006327.s007.tif]

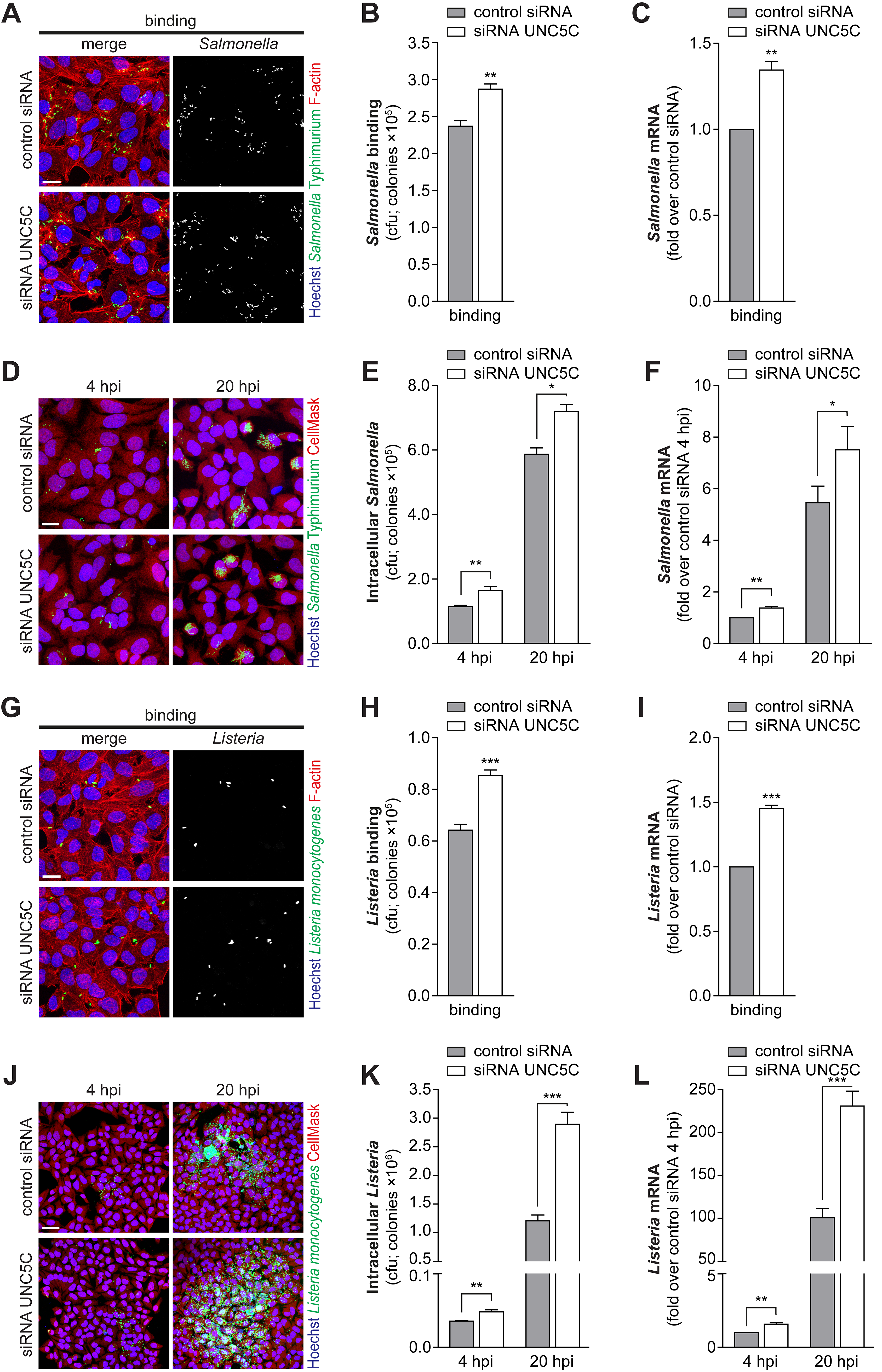

Supplement: S8 Fig — A-F. Representative images (A, D), cfu quantification (B, E) and quantification by qRT-PCR (C, F) of Salmonella WT interaction with HeLa cells transfected with UNC5C or control siRNA, at binding (A-C), or 4 and 20 hpi (D-F). G-L. Representative images (G, J), cfu quantification (H, K) and Listeria quantification by qRT-PCR (I, L) of HeLa cells infected with Listeria WT, upon transfection with UNC5C or control siRNA, and analyzed at three times post-infection: binding (G-I) or 4 and 20 hpi (J-L)). Salmonella and Listeria infection were performed at MOI 50 for binding and MOI 25 for intracellular bacterial load (4 and 20 hpi). For A, D and G, scale bar, 20 μm; for J, 50 μm. Results are shown as mean ± s.e.m. from 5 independent experiments; *P<0.05, **P<0.01, ***P<0.001. (TIF) [file ppat.1006327.s008.tif]

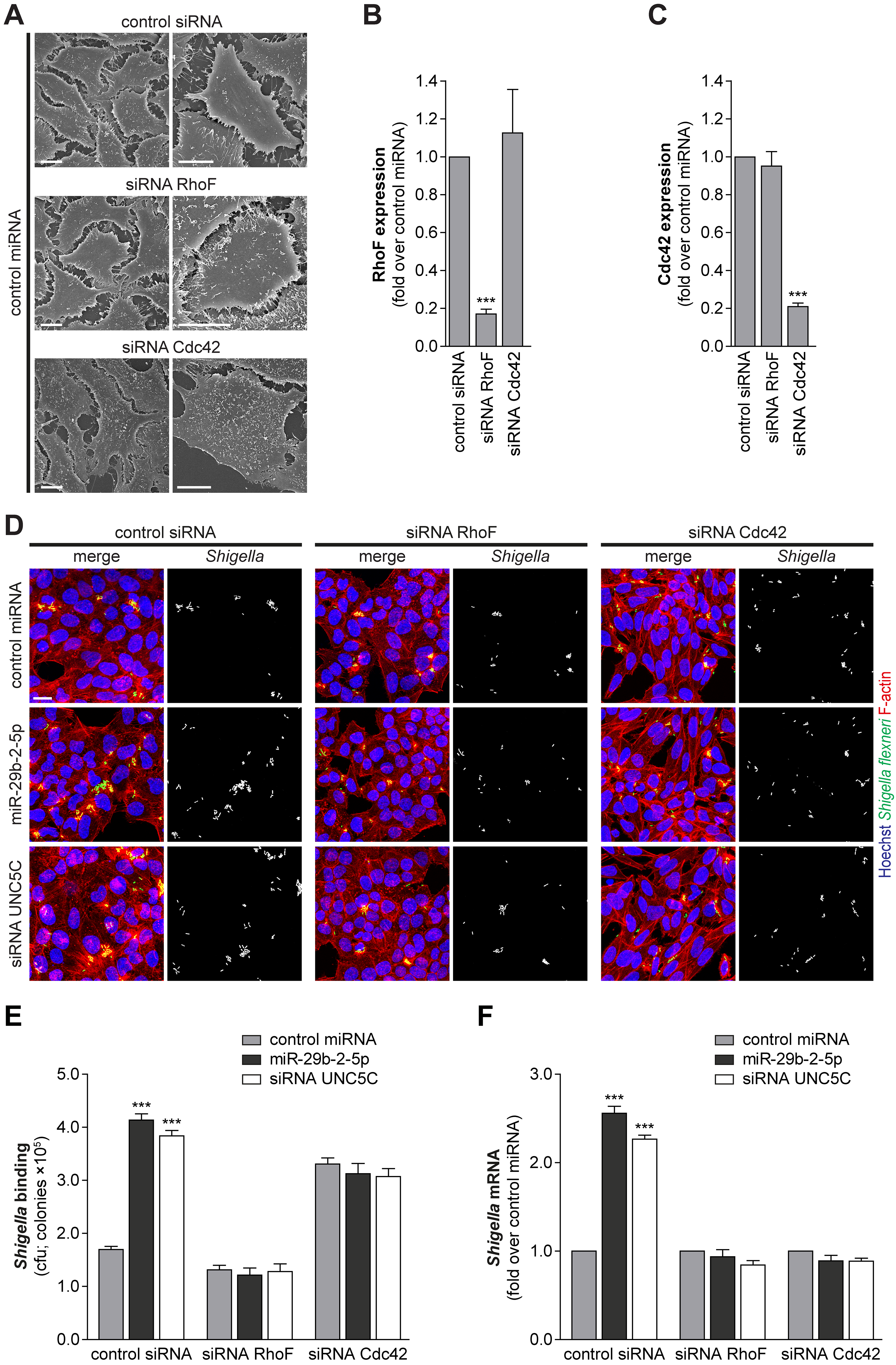

Supplement: S9 Fig — A. Scanning electron microscopy of HeLa cells co-transfected with control miRNA and control, RhoF or Cdc42 siRNAs. Images obtained at lower (left) and higher (right) magnifications are shown for each treatment. Scale bar, 10 μm. Representative images of the scanning electron microscopy were selected from 3 independent experiments. B and C. RhoF (B) and Cdc42 (C) expression in HeLa cells transfected with RhoF, Cdc42 and control siRNAs. Results are shown normalized to cells transfected with control siRNA. D–F. Representative images (D), cfu quantification (E) and qRT-PCR quantification (F) of Shigella binding to HeLa cells co-transfected with RhoF, Cdc42 or control siRNAs and UNC5C siRNA, miR-29b-2-5p or control miRNA mimics. Scale bar, 20 μm. Results are shown as mean ± s.e.m. from 5 independent experiments, normalized to control miRNA (panel F) or control siRNA (panels B and C); ***P<0.001. (TIF) [file ppat.1006327.s009.tif]
