# Supplementary material for: Analysis of host microRNA function uncovers a role for miR-29b-2-5p in Shigella capture by filopodia
Source: PLoS Pathog. 2017 Apr 10;13(4):e1006327. doi: 10.1371/journal.ppat.1006327 (PMC5398735; doi:10.1371/journal.ppat.1006327)
Supplement: S1 Table — Results of the percentage of Shigella infected cells for the top 10 miRNAs increasing infection identified in the microscopy-based high-throughput screening of a genome-wide library of miRNA mimics. Results of a Salmonella screening, previously published by our group (Maudet et al. 2014), are shown for comparison. Of note, the mature miR-365a-3p and miR-365b-3p have the same sequence; in miRBase 13, this miRNA was named miR-365 (result is shown in italic). Results for percentage of infected cells are shown normalized to control miRNA. (PDF) [file ppat.1006327.s010.pdf]

this study					Maudet et al 2014				
id	sc1	sc2	sc3	%inf cells avg fold over control	id	sc1	sc2	sc3	%inf cells avg fold over control
hsa-miR-3177-3p	7,04	4,34	4,16	5,18	not available in miRBase13				
hsa-miR-9-5p	5,93	4,24	3,90	4,69	hsa-miR-9	0,66	0,83	0,44	0,64
hsa-miR-365a-3p	5,79	3,90	3,42	4,37	*hsa-miR-365*	*2,21*	*1,80*	*1,43*	*1,81*
hsa-miR-3661	5,44	3,77	3,32	4,18	not available in miRBase13				
hsa-miR-107	5,44	3,53	3,34	4,10	hsa-miR-107	1,23	1,25	0,74	1,08
hsa-miR-4675	4,92	3,38	3,58	3,96	not available in miRBase13				
hsa-miR-1285-3p	4,47	3,65	3,25	3,79	hsa-miR-1285	1,43	1,37	0,83	1,21
hsa-miR-29b-2-5p	4,56	3,68	3,00	3,75	hsa-miR-29b-2*	1,11	0,99	0,78	0,96
hsa-miR-365b-3p	4,58	3,40	3,12	3,70	*hsa-miR-365*	*2,21*	*1,80*	*1,43*	*1,81*
hsa-miR-4788	5,31	2,40	3,22	3,64	not available in miRBase13				
