# Supplementary material for: Analysis of host microRNA function uncovers a role for miR-29b-2-5p in Shigella capture by filopodia
Source: PLoS Pathog. 2017 Apr 10;13(4):e1006327. doi: 10.1371/journal.ppat.1006327 (PMC5398735; doi:10.1371/journal.ppat.1006327)
Supplement: S2 Table — List of 52 genes that are both repressed by miR-29b-2-5p (≥ 1.5-fold down-regulation; reads ≥ 25 for cells transfected with control miRNA) and have increased expression in Shigella infected cells (6 hpi; ≥2-fold up-regulation Shigella + population relative to mock; ≤ 2-fold change Shigella - population relative to mock; reads ≥25 for mock). (PDF) [file ppat.1006327.s011.pdf]

gene	Gene id	fold change in miR-29-2-5p vs control miR	fold change in Shigella+ vs mock	comments
ABLIM3	22885	0,33	3,41	
ADAMTS15	170689	0,34	2,58	
AKAP2	11217	0,64	2,54	siRNA not in library
AKAP5	9495	0,52	2,39	
ANKLE2	23141	0,65	2,15	siRNA not in library
ANXA8L1	653145	0,10	2,45	
ANXA8L2	414306	0,12	2,47	replaced by ANXA8L1 in NCBI database
ARHGEF40	55701	0,47	2,16	siRNA not in library
ATP8B1	5205	0,57	3,53	
BCL3	602	0,40	2,38	
BCL6	604	0,22	2,40	
BDKRB2	624	0,45	2,28	
BTG2	7832	0,41	2,11	
CCDC135	84229	0,59	14,39	
CDKN2B	1030	0,38	2,69	
CLIC4	25932	0,59	2,06	
COL4A2	1284	0,61	2,26	
COL7A1	1294	0,25	2,08	
EHD1	10938	0,59	2,04	
EPPK1	83481	0,34	3,00	
FAM110B	90362	0,29	4,38	
FBLN5	10516	0,51	2,52	
FHL3	2275	0,55	2,09	
FOSL2	2355	0,47	2,46	
FSTL3	10272	0,43	2,58	
GADD45B	4616	0,53	7,22	
GPR153	387509	0,66	2,14	
HCN3	57657	0,61	2,16	
HDAC10	83933	0,41	9,17	
HRH1	3269	0,39	2,02	
LOXL2	4017	0,22	2,04	
MAFB	9935	0,17	2,28	
MEX3B	84206	0,66	3,14	siRNA not in library
MICALL2	79778	0,55	2,11	
NIPAL4	348938	0,10	2,56	siRNA not in library
PALLD	23022	0,53	2,04	
PALM2-AKAP2	445815	0,64	2,54	
PHLDA3	23612	0,53	3,54	
PIM1	5292	0,55	2,39	
PRR24	255783	0,38	2,22	siRNA not in library
RIPK4	54101	0,27	2,32	
SH3RF2	153769	0,58	2,86	
SIPA1L2	57568	0,45	2,12	
SNAI2	6591	0,24	2,25	
SYNGR3	9143	0,46	3,03	
TEF	7008	0,31	2,16	
TRIB1	10221	0,54	3,35	
UCN2	90226	0,24	2,68	
ULBP2	80328	0,28	2,25	
UNC5C	8633	0,50	3,93	
VAV3	10451	0,37	3,62	
ZCCHC24	219654	0,52	2,36	
ZNF408	79797	0,53	2,66	
