# Supplementary material for: Analysis of host microRNA function uncovers a role for miR-29b-2-5p in Shigella capture by filopodia
Source: PLoS Pathog. 2017 Apr 10;13(4):e1006327. doi: 10.1371/journal.ppat.1006327 (PMC5398735; doi:10.1371/journal.ppat.1006327)
Supplement: S3 Table — Percentage of HeLa cells infected with Shigella WT upon treatment siRNAs targeting 46 genes. SiRNAs that decreased cell viability to less than 65% of control were excluded from further analysis and are highlighted in gray. Results for percentage of infected cells and cell viability are shown normalized to control siRNA. (PDF) [file ppat.1006327.s012.pdf]

gene	geneid	% inf cells (fold over control) replicate 1	% inf cells (fold over control) replicate 2	% inf cells (fold over control) replicate 3	% inf cells avg fold over control	avg cell number (fold over control)
UNC5C	8633	4,78	3,45	3,95	4,06	1,27
CLIC4	25932	4,41	2,29	3,92	3,54	0,40
ZCCHC24	219654	3,86	3,08	3,28	3,40	1,15
BDKRB2	624	3,08	2,06	4,06	3,07	0,59
MAFB	9935	3,25	2,61	2,95	2,94	1,39
SH3RF2	153769	3,14	2,49	3,13	2,92	0,68
VAV3	10451	3,57	2,61	2,57	2,92	1,29
PALLD	23022	2,14	2,57	3,15	2,62	1,66
EHD1	10938	2,23	2,05	3,11	2,46	0,92
ZNF408	79797	2,68	1,70	2,95	2,45	0,79
PALM2-AKAP2	445815	2,63	1,45	3,22	2,43	0,42
BCL3	602	1,81	2,30	3,15	2,42	0,88
ADAMTS15	170689	2,06	1,87	2,93	2,29	1,18
AKAP5	9495	2,05	1,59	3,20	2,28	0,95
SYNGR3	9143	3,21	1,83	1,68	2,24	0,86
FHL3	2275	2,03	1,56	2,93	2,17	0,99
FBLN5	10516	2,15	1,77	2,59	2,17	0,54
FSTL3	10272	2,37	1,11	2,89	2,12	0,34
SNAI2	6591	2,66	1,53	2,08	2,09	0,63
HDAC10	83933	2,11	1,72	2,41	2,08	0,63
GADD45B	4616	2,78	1,23	2,15	2,05	1,27
BCL6	604	2,01	1,25	2,87	2,04	0,52
TEF	7008	1,33	1,44	3,12	1,96	1,09
CDKN2B	1030	1,70	1,75	2,41	1,95	1,28
FAM110B	90362	1,62	1,18	2,93	1,91	0,49
PHLDA3	23612	1,82	1,46	2,22	1,84	0,65
FOSL2	2355	1,72	1,43	2,29	1,81	1,01
MICALL2	79778	2,47	1,20	1,69	1,79	0,70
HRH1	3269	1,30	1,42	2,42	1,71	0,78
TRIB1	10221	1,84	1,18	2,11	1,71	0,97
BTG2	7832	1,82	1,13	2,16	1,70	1,29
EPPK1	83481	1,55	1,46	1,97	1,66	1,65
SIPA1L2	57568	2,01	0,90	1,85	1,58	0,76
RIPK4	54101	1,69	0,76	1,99	1,48	0,60
COL7A1	1294	1,83	0,86	1,49	1,39	1,54
CCDC135	84229	0,77	0,39	2,91	1,36	0,56
ANXA8L1	653145	1,58	1,08	1,35	1,34	1,23
GPR153	387509	0,89	0,57	2,37	1,28	0,76
HCN3	57657	1,52	0,91	1,37	1,27	1,35
PIM1	5292	1,40	0,94	1,35	1,23	1,08
LOXL2	4017	0,77	0,30	2,40	1,16	0,26
ATP8B1	5205	0,91	0,91	1,47	1,09	0,76
UCN2	90226	1,42	0,62	1,07	1,04	0,80
ULBP2	80328	0,97	0,51	1,50	0,99	0,81
ABLIM3	22885	1,02	0,13	1,07	0,74	0,21
COL4A2	1284	0,53	0,20	1,26	0,66	0,63
